# The Olfactory Organ Is Populated by Neutrophils and Macrophages During Early Development

**DOI:** 10.3389/fcell.2020.604030

**Published:** 2021-01-18

**Authors:** M. Fernanda Palominos, Kathleen E. Whitlock

**Affiliations:** Programa Doctorado en Neurociencia, Facultad de Ciencia, Centro Interdisciplinario de Neurociencia de Valparaíso (CINV), Instituto de Neurociencia, Universidad de Valparaíso, Valparaíso, Chile

**Keywords:** nervous system, microglia, zebrafish, vasculature, neuron, macrophage, olfactory, immune

## Abstract

The immune system of vertebrates is characterized by innate and adaptive immunity that function together to form the natural defense system of the organism. During development innate immunity is the first to become functional and is mediated primarily by phagocytic cells, including macrophages, neutrophils, and dendritic cells. In the olfactory sensory system, the same sensory neurons in contact with the external environment have their first synapse within the central nervous system. This unique architecture presents a potential gateway for the entry of damaging or infectious agents to the nervous system. Here we used zebrafish as a model system to examine the development of the olfactory organ and to determine whether it shares immune characteristics of a host defense niche described in other tissues. During early development, both neutrophils and macrophages appear coincident with the generation of the primitive immune cells. The appearance of neutrophils and macrophages in the olfactory organs occurs as the blood and lymphatic vascular system is forming in the same region. Making use of the neurogenic properties of the olfactory organ we show that damage to the olfactory sensory neurons in larval zebrafish triggers a rapid immune response by local and non-local neutrophils. In contrast, macrophages, although present in greater numbers, mount a slower response to damage. We anticipate our findings will open new avenues of research into the role of the olfactory-immune response during normal neurogenesis and damage-induced regeneration and contribute to our understanding of the formation of a potential host defense immune niche in the peripheral nervous system.

## Introduction

The olfactory sensory system is composed of the peripheral olfactory epithelium (OE), where the continually renewing olfactory sensory neurons (OSNs) are located. The axons of the OSNs reach the central nervous system (CNS) via the olfactory nerve (ON), where they make their first synapses in the olfactory bulb (OB) (Sakano, 2010; Whitlock, 2015). Thus, unlike other sensory systems, the first synapses of the OSNs lie within the CNS. This unique organization creates a potential pathway for chemical or biological agents to enter the CNS. Yet, although pathogens can enter the CNS via the OE (Pägelow et al., 2018), it is striking that our brains are not besieged by infections that enter through this direct olfactory portal.

Recently, it has been shown that in mammals “host defense niches” exist where myeloid cell types, such as neutrophils, remain associated with the tissue instead of patrolling the body (Yipp et al., 2017). These resident cells have been described in the lungs, a tissue that like the olfactory epithelia comes in contact with potential damaging airborne substances. In mammals, the airways of the nose and mouth have a network of lymphoid tissue in the pharynx and palate (tonsils), called nasopharynx-associated lymphoid tissue, which protect against invasion by neurotropic microorganisms, including viruses. Like mammals, zebrafish have the basic myeloid cell types including monocytes, neutrophils, eosinophils, mast cells, and dendritic cells, yet they do not have organized lymphoid structures such as tonsils/lymph nodes. Limited studies suggest that fish have a diffuse network of lymphoid and myeloid cells associated with the olfactory organ (OO) that may resemble mucosal immune tissues (Tacchi et al., 2014).

Similar to mammals, zebrafish generate blood/immune cells in successive waves during development. In zebrafish, during the first phase of hematopoiesis, precursors arise from the mesoderm generating the rostral blood island (RBI) and intermediate cell mass (ICM) before entering the circulation (Davidson and Zon, 2004). Myeloid cell precursors including monocytes/macrophages and granulocytes develop by 12 h postfertilization (hpf) (Lieschke et al., 2002), and functional macrophages and neutrophils are present by 30 hpf (Herbomel et al., 1999; Le Guyader et al., 2008). The RBI will also give rise to larval microglia via primitive macrophages (Herbomel et al., 1999, 2001; Xu et al., 2015). The larval zebrafish has been used to study immune system development and function because of the optical clarity, availability of reporter lines expressed in immune cell types, and sequenced genome (Mathias et al., 2006; Renshaw et al., 2006; Hall et al., 2007). Because of the regenerative properties of fish, in tissues such as the tail and fins, zebrafish are readily amenable to wounding studies (induced by cutting the tail for example) where the response of the innate immune system can be visualized and manipulated in intact living animals (Mescher et al., 2017). Here we make use of the peripheral olfactory sensory system to explore the early development of immune cells types and their potential association with the OO.

Previously, through microarray and RNAseq analyses of adult OE zebrafish (Harden et al., 2006; Calfun et al., 2016), we found that, in addition to OE specific genes, genes normally expressed in both the innate and the adaptive immune systems were also expressed. These findings prompted us to investigate the potential “immune architecture” of the OE. Because of the early development of innate immune system (Masud et al., 2017), we investigated the presence and dynamics of neutrophils and macrophages in the olfactory sensory system of developing zebrafish to better characterize the immune cells as well as understand their potential response to damage in the developing OO.

## Materials and Methods

### Animals

Zebrafish were maintained in a recirculating system (Aquatic Habitats Inc., Apopka, FL) at 28°C on a light–dark cycle of 14 and 10 h, respectively. All fish were maintained in the Whitlock Fish Facility at the Universidad de Valparaiso. Wild-type fish of the Cornell strain (derived from Oregon AB) were used. All protocols and procedures employed were reviewed and approved by the Institutional Committee of Bioethics for Research With Experimental Animals, University of Valparaiso (#BA084-2016). Embryos were obtained from natural spawnings in laboratory conditions and raised at 28.5°C in embryo medium as previously described (Westerfield, 2007). Staging was done according to Kimmel et al. (1995). At 5 days postfertilization (dpf), larvae were transferred to finger bowls and fed daily with Larval AP100 dry diet (Zeigler®) until processed. Larvae were defined as ranging from 3 to 14 dpf, and 21 dpf animals were considered as juveniles. Transgenic lines were used to visualize specific cell types. *Tg(BACmpx:gfp)i114, Tg(mpx:GFP) Tg(mpx:EGFP)*, (Renshaw et al., 2006); (Tg(fli1a:EGFP)y1, Tg(fli1a:EGFP), (Lawson and Weinstein, 2002); *Tg(*−*5.2lyve1b:DsRed)*^*nz*101^, *Tg(2lyve1b:DsRed) Tg(*−*5.2lyve1b:EGFP)*^*nz*151^ *Tg(lyve1b:EGFP)*, (Okuda et al., 2012); *Tg(gata1a:DsRed)*^*sd*2^, *Tg(gata1a:DsRed)* (Traver et al., 2003); *Tg(pOMP2k:gap-YFP)*^*rw*032*a*^, *(OMP:YFP), Tg(pOMP2k:lyn-mRFP)*^*rw*035*a*^, *Tg(OMP:RFP*), *Tg(pTRPC4.5k:gap-Venus)*^*rw*037*a*^ (Sato et al., 2005); *Tg(mpeg1:mCherry)* (Ellett et al., 2011); and *Tg(lysC:DsRED2)*, (Hall et al., 2007).

### Copper Exposure

Initial dose–response analysis was performed based on previous work in zebrafish and salmon (Baldwin et al., 2003; Hernandez et al., 2011). A stock solution of 10 mM CuSO_4_ was diluted in filtered embryo medium (Westerfield, 2007) for a final concentration of 10 μM CuSO_4_. Staged larvae were exposed to 10 μM CuSO_4_ for 4 h and then washed out. The long-term effects of copper on neutrophil movement to the OO were quantified in individual larvae using adapted ChIn assay (d'Alençon et al., 2010).

### Immunocytochemistry and Cell Labeling

Staged larvae were fixed in 4% paraformaldehyde (PFA) in 0.1 M phosphate buffer, pH 7.3, or 1× phosphate-buffered saline (PBS) pH 7.3. Larvae were rinsed three times in phosphate buffer or PBS, permeabilized in acetone at −20°C for 10 min and then incubated for 2 h in blocking solution [10 mg/mL bovine serum albumin, 1% dimethyl sulfoxide (DMSO), 0.5% Triton X-100 (Sigma), and 4% normal goat serum in 0.1 M phosphate buffer or 1× PBS]. Primary antibodies used were anti-RFP (rabbit 1:250, Life Technologies), anti-GFP (mouse 1:500, Life Technologies), anti-GFP (rabbit 1:500, Invitrogen), anti-SOX2 (mouse 1:250, Abcam), anti-DsRed (mouse 1:500, Santa Cruz Biotechnology), and anti-HuC/D (rabbit 1:500, Invitrogen). Larvae up to 14 days were incubated in primary antibodies for 3 to 4 days. After washes, tissues were incubated overnight in any of the following secondary antibodies as appropriate: Dylight 488–conjugated anti–mouse antibody (goat 1:500, Jackson ImmunoResearch), Alexa Fluor 488–conjugated anti–rabbit antibody (goat 1:1,000, Molecular Probes), Alexa Fluor 568 conjugated anti–rabbit antibody (goat 1:1,000, Molecular Probes), Alexa Fluor 568 conjugated anti-mouse antibody (goat 1:1,000, Molecular Probes), and Dylight 650 conjugated anti–rabbit antibody (goat 1:500, Jackson ImmunoResearch). Tissues were then rinsed in 0.1 M phosphate buffer or 1× PBS with 1% DMSO, stained for DAPI (1 μg/mL, Sigma), washed in 0.1 M phosphate buffer or 1× PBS and mounted in 1.5% low melting temperature agarose (Sigma) in an Attofluor Chamber for subsequent imaging (see below).

### Cryosectioning

Seven-dpf larvae were sacrificed and then fixed and embedded in 5% sucrose/1.5% agarose in mqH_2_O. Blocks were then submerged in 30% sucrose for 2 to 3 days and then stored covered by O.C.T. Compound (Tissue-Tek®) in cryomolds at −20°C. Twenty-five-micrometer cryosections were processed for immunofluorescense as described above; primary and secondary antibodies were incubated overnight.

### TUNEL Labeling

Larvae were processed using *in situ* Cell Death Detection Kit, Fluorescein (Roche), according to manufacturer recommendations. Briefly, larvae were permeabilized for 1 h at 37°C, washed twice, and labeled at the same temperature for 1 h. DAPI staining was used for nuclear labeling. Larvae were mounted in 2% low melting temperature agarose (Sigma) in an Attofluor Chamber for imaging (see below). Fluorescent signals in TUNEL-labeled preparations were quantified by mean pixel intensities from green (fluorescein from TUNEL staining), green (GFP from *trpc2:GFP*), and red (RFP from *OMP:RFP*) in OE and OB (selected as different ROIs in FIJI). Values were normalized by mean pixel intensity of the DAPI stained whole head (as another ROI).

### Microscopy

Fluorescent images were acquired using a Spinning Disc microscope Olympus BX-DSU (Olympus Corporation, Shinjuku-ku, Tokyo, Japan) with ORCA IR2 Hamamatsu camera (Hamamatsu Photonics, Higashi-ku, Hamamatsu City, Japan) and Olympus CellR software (Olympus Soft Imaging Solutions, Munich, Germany) or confocal laser scanning microscope (Nikon C1 Plus; Nikon, Tokyo, Japan). Images were deconvoluted in AutoQuantX 2.2.2 (Media Cybernetics, Bethesda, MD, USA) and processed using FIJI (National Institutes of Health, Bethesda, Maryland, USA; (Schindelin et al., 2012) and CellProfiler (McQuin et al., 2018).

### Live Imaging

For live imaging of the olfactory sensory system, larvae were anesthetized (2% Tricaine Sigma) mounted in a cut tip of plastic Pasteur pipette in 2% low temperature agarose (Sigma) in embryo medium (Westerfield, 2007). The larvae were imaged in frontal view in an Attofluor Chamber (Thermo Fisher Scientific) filled with Embryo medium. The agarose covering the olfactory system was removed. Temperature was maintained at 26–28°C, and images were captured using a Spinning disc confocal microscope (Olympus) with a 20 × 0.95 NA water immersion LUMPlanFL/IR objective.

Time-lapse videos of copper exposure: To generate the time-lapse movies (**Figures 5**, **6**, **8**, **9**), stacks of images were collected with 3 μm/optical section in a total depth of 150-μm depth. All tracking data from time-lapse microscopy in control and copper-exposed larvae were processed using MTrackJ tracker in FIJI. Chemotactic index (CI) was calculated as described by Lämmermann et al. (2013), taking left or right OO as reference. Briefly, CI was defined as cos(α) with α as the angle between the distance vector to the damage site (OO) and the actual movement vector.

### Image Analyses

For analysis of neutrophils and macrophages: Only cells within the boundaries of the sensory tissue were counted, and the values were given as the average of total number of mpx:GFP-positive or mpeg1:mCh-positive in both OOs with standard deviation. Values given for paired sensory structure are a sum of the individual sensory tissues. For time-lapse videos, all counts of neutrophils and macrophages in the two OOs were combined for each animal and the mean/SEM calculated for each time point.

### Statistics

Data are presented as means ± standard deviations. Experiments number and statistical analysis were done using Prism 6 (GraphPad) and are indicated in each figure legend. Unpaired Student *t*-tests were performed unless otherwise indicated. P-values are indicated as follows: ^*^P < 0.05, ^**^P < 0.01, ^***^P < 0.001, ^****^P < 0.0001.

## Results

### Phagocytic Cell Populations in the Developing Olfactory Organ

**Figure 1:**
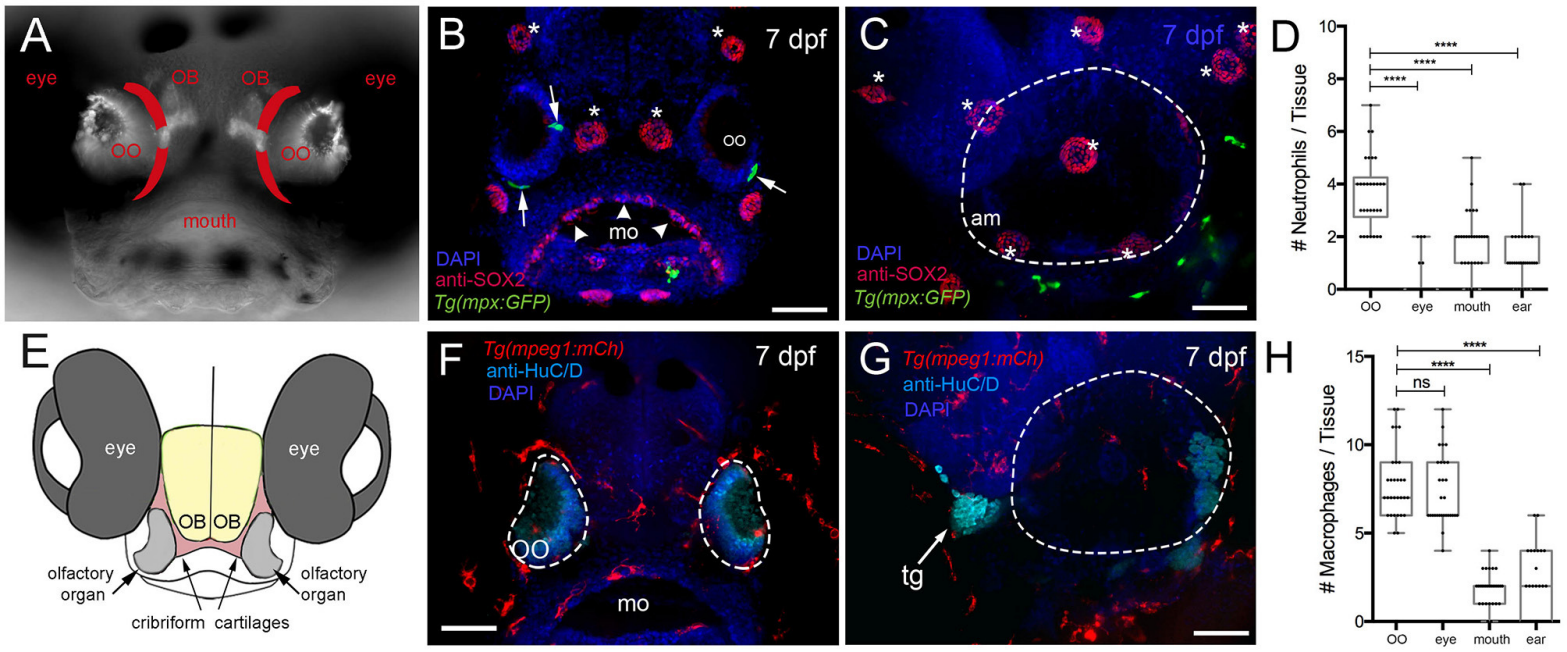
Neutrophils and macrophages are found in developing sensory systems. **(A)** Frontal view of 7-dpf larvae. Bright-field/fluorescent image in whole-mount larvae with OMP:RFP+ OSNs. Lines in red indicate the future adult cribriform plate. **(B)** Frontal view, whole-mount 7-dpf *Tg(mpx:GFP)* larva, anti-Sox2–positive taste buds (red, arrowheads), neuromasts (red, asterisks), and neutrophils (green). **(C)** Lateral view, whole-mount 7 dpf larva. Neutrophils (green) associated with border of the ear (dashed line) (am: ampulla). **(D)** Neutrophils are highly represented in the olfactory organ (OO) with fewer neutrophils associated with the gustatory and auditory systems. No neutrophils were observed in the retina (*n* = 30 animals, one-way ANOVA, Tukey test, P < 0.0001). **(E)** Diagram of head of larva showing generalized position of the cartilages (red) giving rise to the cribriform plate in the adult animal. **(F)** Frontal view, whole-mount 7-dpf *Tg(mpeg1:mCh)* larvae, anti-HuC/D–positive neurons in the OO (pale blue), macrophages (red). **(G)** Lateral view, whole-mount 7 dpf. Macrophages (red) associated with border of the ear (dashed line). **(H)** The olfactory organ (OO) and the eye have the most macrophages, with fewer macrophages associated with gustatory (mo) and auditory (ear) systems (*n* = 30 animals, one-way ANOVA, Tukey test, P < 0.001, ns = non-significant). DAPI: blue, mo: mouth, tg: trigeminal ganglia. Scale bars: **(B,C,F,G)** = 100 μm.

We first quantified phagocytic cells (neutrophils and macrophages) of the immune system to determine whether they were present in peripheral sensory systems during early development. We used the *Tg(mpx:GFP)* line to visualize neutrophils, a leukocyte subtype with strong myeloperoxidase (mpx) activity, and the *Tg(mpeg1:mCh)* line (macrophage-expressed gene, *mpeg1.1*, encodes perforin-2, a pore-forming protein associated with host defense against pathogens) to visualize macrophages in fixed whole-mount larvae (Figure 1). Olfactory sensory structures do not appear as a stratified epithelium until later in development; thus, we refer to the tissue as an OO (Figures 1A,E). At 7 dpf mpx:GFP+ neutrophils were found associated with the OOs (Figure 1B, green, arrows) and anti-Sox2–positive taste buds (Figure 1B, arrowheads). In contrast few neutrophils were directly associated with the ear (Figure 1C, green). When quantifying neutrophils in the developing sensory systems (Figure 1D), the olfactory sensory system has more neutrophils than other sensory systems (*n* = 30 animals per sensory system, one-way ANOVA, Tukey test, P < 0.05). No neutrophils were observed in the retina. In contrast to neutrophils, at 7 dpf there were many more macrophages in the OOs (Figure 1F), but not in the ear (Figure 1G). Unlike the situation with neutrophils, the OO and eye had equal numbers of macrophages (Figure 1H), yet the gustatory (mouth) and auditory (ear) number remained lower (*n* = 30 animals per sensory system, one-way ANOVA, Tukey test, P < 0.05).

**Figure 2:**
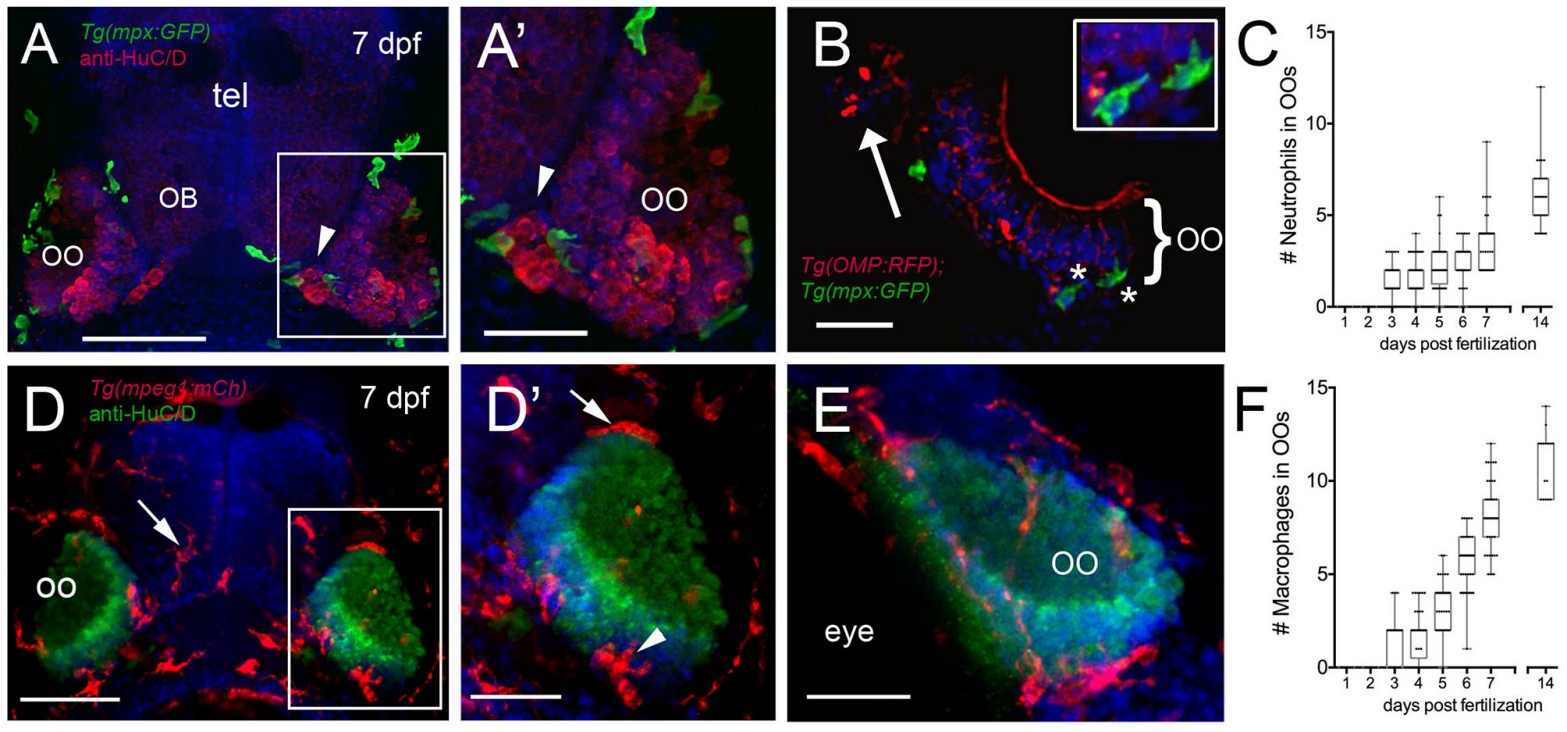
Neutrophils and macrophages populate the olfactory system of juvenile animals. **(A)** Frontal view of *Tg(mpx:GFP)* 7dpf larva with anti-HuC/D–positive (red) neurons in the olfactory organ (OO) adjacent to the olfactory bulb (OB). **(A′)** Image of OO (boxed area in **A**) with neutrophils (green). **(B)** 25 μm cryosection of a 7-dpf *Tg(OMP:RFP);Tg(mpx:GFP)* larva neutrophils (green, asterisks) localized within the OO margin and adjacent to OSNs (red, see inset). **(C)** Average number (±SEM) of neutrophils in OOs of *Tg(mpx:GFP)* during the first 2 weeks postfertilization (*n* = 45 larvae). **(D)** Frontal view of *Tg(mpeg1:mCh)* 7 dpf larva. Anti-HuC/D–positive (green) neurons populate the olfactory organs (OO). **(D′)** Image of OO (boxed area in **D**) with macrophages (red) adjacent to the OO (arrow) and within the OO (arrowhead). **(E)** Lateral oblique view of OO at 7 dpf. **(F)** Average number (±SEM) of macrophages in OOs of *Tg(mpeg1:mCh)* during the first 2 weeks post-fertilization (*n* = 45 larvae). Scale bars: **(A,D)** = 100 μm; **(A′,D′,E)** = 50 μm, **(B)** = 25 μm.

We next quantified the number of mpx:GFP+ neutrophils (Figures 2A–C) and mpeg1:mCh+ macrophages (Figures 2D–F) associated with the OOs during early development. At 3 dpf, neutrophils started to appear associated with the OOs (Figure 2C; 1.4 ± 0.1), and by 7 dpf (Figures 2A,B, green), there was an average of 3.6 ± 0.2 (Figure 2C), and neutrophil numbers increased steadily through the first 2 weeks (Figure 2C; 6.0 ± 0.3). Like neutrophils, at 3 dpf, macrophages started to appear associated with the OOs (Figure 2F; 1.1 ± 0.1), and by 7 dpf (Figures 2D,D′,E, red), there was an average of 8.2 ± 0.2 macrophages (Figure 2F). Macrophage numbers increased steadily through the first 2 weeks (Figure 2F, 11 ± 0.4).

### Blood Lymphatic System in the Developing Olfactory Organ

**Figure 3:**
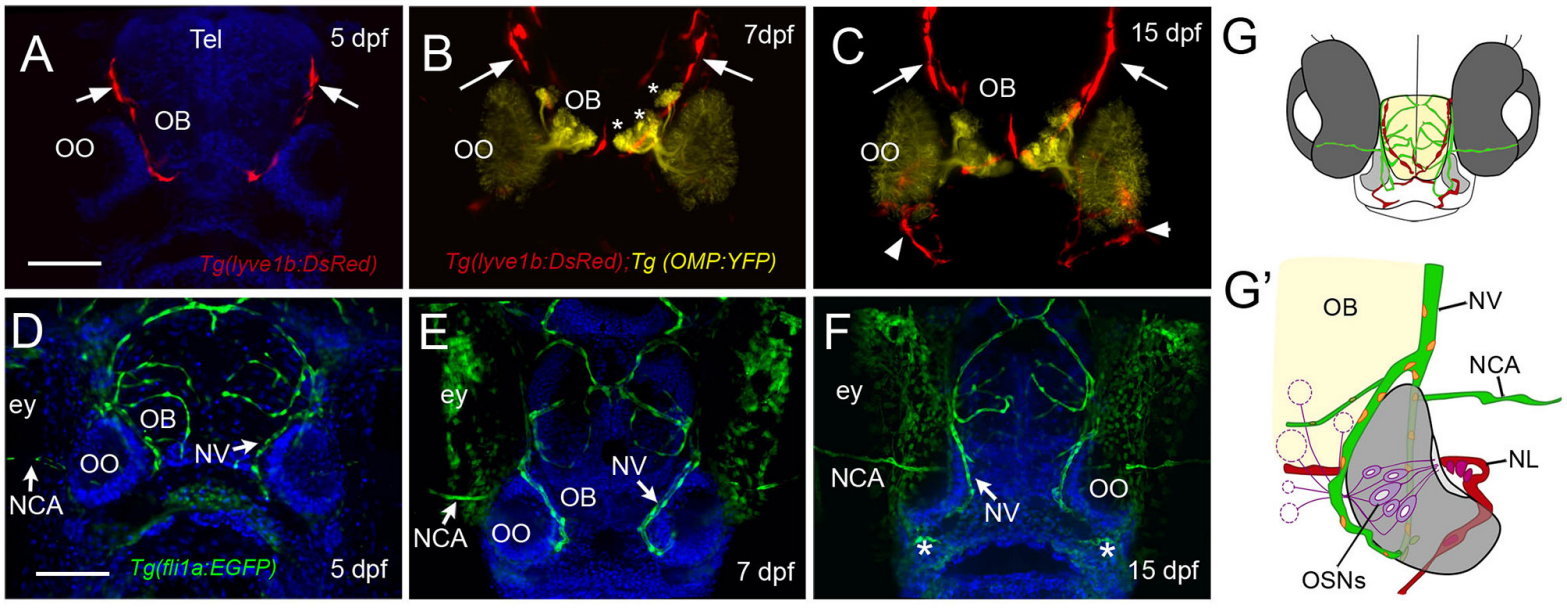
The developing olfactory organs have an extensive blood-lymphatic system. Lyve1b:DsRed+ lymphatic vessels (red) at 5 dpf **(A)**, 7 dpf **(B)**, and 15 dpf **(C)**. **(A–C)** Lyve1b:DsRed+ lymphatic vasculature (red, arrows) extends from the dorsal brain toward the olfactory organs (OO). **(B,C)** Lymphatic vessels extend to region of olfactory sensory neurons (OMP:YFP+), yellow, asterisks) in olfactory bulb (OB). **(C)** At 15 hpf, nasal lymphatic vasculature (red, arrowheads) is now visible wrapping around the posterior OO and associating with the ventral lateral OOs **(G)**. Fli1a:EGFP+ blood vasculature (green) at 5 dpf **(D)**, 7 dpf **(E)**, and 15 dpf **(F)**. Blood vessels (green) forming nasal vein (NV) and nasal ciliary artery (NCA) are present at 5 dpf. The NV extends ventrally **(E)** encircling the OO **(F)**. **(G)** Diagram of head of 15 dpf larva showing telencephalon (green) and olfactory organs (gray). **(G′)** Olfactory organ (gray) summarizing blood (green) and lymphatic (red) vasculature. Nuclei of NV (orange) are positive for both lyve1b:DsRed and fli1a:EGFP. **(A,D,E,F)**: DAPI (blue). OO: olfactory organ, OB: olfactory bulb, ey: eyes. Scale bars: **(A–C)** = 200 μm, **(D–F)** = 200 μm.

Recently, the lymphatic vasculature (LV) of the zebrafish brain has been described (Bower et al., 2017; van Lessen et al., 2017; Bower and Hogan, 2018), but little is known about the developing blood vasculature (BV) and LV associated with the olfactory sensory system. Using the *Tg(lyve1b:DsRed); Tg(OMP:YFP)* double-transgenic line at 5 dpf, we found LV on the dorsal–lateral surface of the telencephalon (Figure 3A, red, arrows) extending around the region of the forming OB. By 7 dpf, the LV encircled the OB region (Figure 3B, OB) where the axons of the OSNs terminate (Figure 3B, asterisks, yellow). At 2 weeks post-fertilization, the dorsal projections were maintained (Figure 3C, red, arrows), and lyve1b:DsRed+ branches were apparent on the ventral side of the OOs (Figure 3C, red, arrowheads, G,G', NL). We visualized the development of BV using the *Tg(fli1a:EGFP)* line (Figures 3D–F, green). At 5 dpf, the BV was already apparent on the dorsal surface of the brain (Figure 3D) and was found associated with the OOs before the LV. The nasal artery/nasal veins (NA/NVs) (Isogai et al., 2001) are the most rostrally projecting of vessels until at least 7 dpf (Figure 3E), with two branches enclosing the OO at 15 dpf (Figures 3F,G,G′, green), a time when the ventral-lateral branch of the LV can be seen entering the OO (Figure 3C, red, arrowhead, G′, NL, red). The nuclei of the NV wrapping along the medial OO (Figure 3G′, orange) were positive for both the LV (lyve1b:DsRed) and the BV marker (fli1a:EGFP), suggesting it is venous–lymphoid in nature. The later developing LV entering the ventral lateral region of the OO (Figure 3C, arrowheads, NL in Figure 3G′, red) expressed only lyve1b:DsRed, suggesting it is differentiated LV.

### Response of Neutrophils and Macrophages to Tissue Damage in the OO

**Figure 4:**
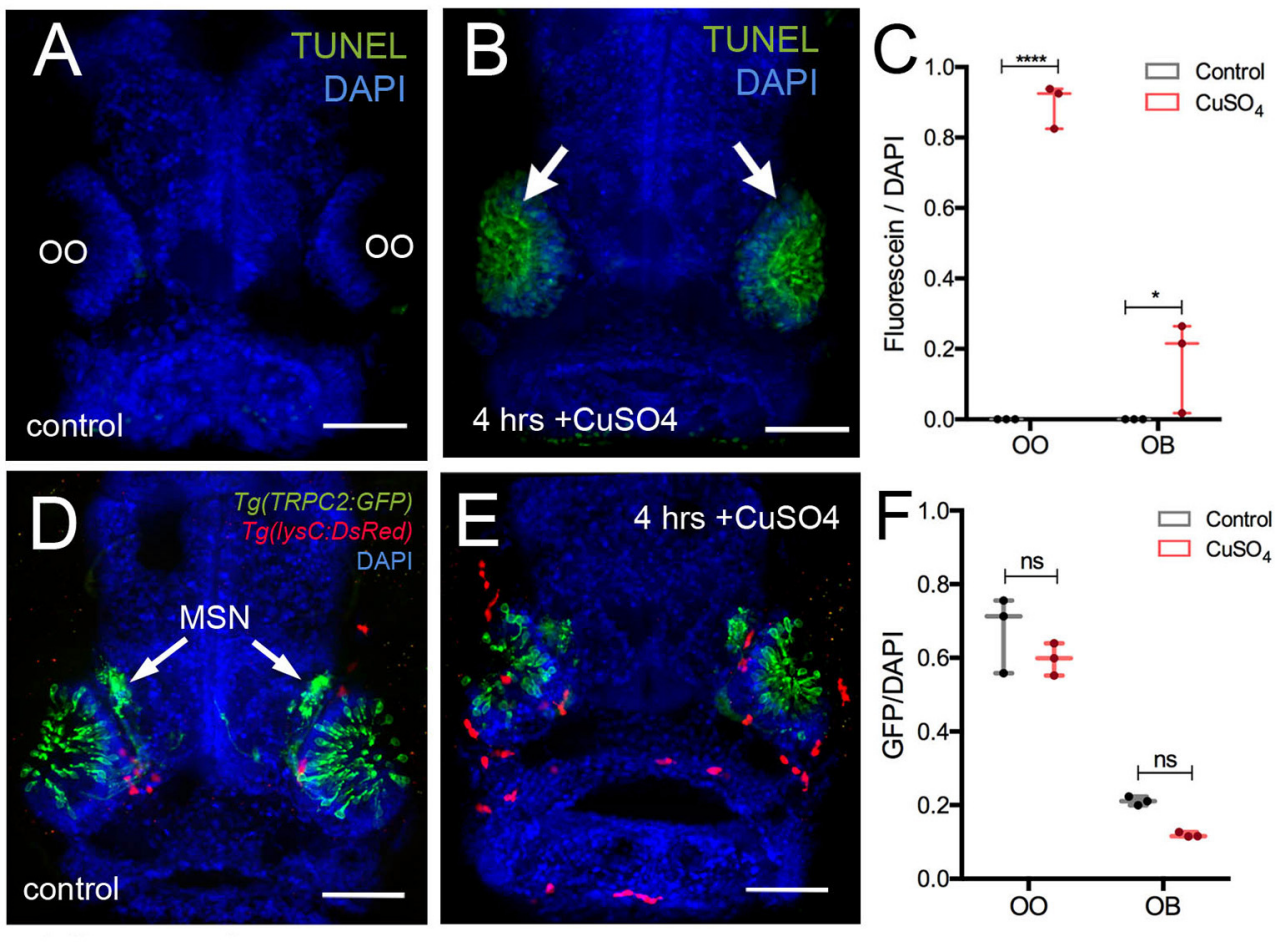
Copper exposure induces cell death and subsequent regeneration in the olfactory sensory system. **(A,B)** TUNEL assay for copper-induced damage to olfactory organ (OO). Whole mount, 5 dpf larva. **(A)** Control fish showed no cell death. **(B)** Only the OOs were positive for TUNEL, green, arrows, DAPI (blue). **(C)** Quantification of TUNEL fluorescence control and treated animals. **(D,E)** Frontal view of *Tg(trpc2:GFP):Tg(lysC:DsRed)* with microvillous sensory neurons (MSN, green) extending into OB in control animals **(D)** and 4 h posttreatment **(E)**. Neutrophils (red) in the OO, but unlike OSNs, microvillous OSNs were largely unaffected. **(F)** Quantification of microvillous OSNs (green, fluorescence) in control (gray) and copper-treated animals (red); no significant decrease in Trpc2:GFP fluorescence was observed. All fluorescence was normalized using DAPI (*n* = 3 larvae, two-way ANOVA, Tukey multiple-comparisons test, ****a = P < 0.0001, b = P < 0.01). All scale bars = 100 μm.

Previously, it has been shown that copper exposure at concentrations ranging from 10^−9^ to 10^−5^ M (Tierney et al., 2010) damages the olfactory sensory epithelia of zebrafish and that the unique neurogenic characteristics of the OE allow for the replacement of the OSNs (Ma et al., 2018). In order to confirm that copper caused cell death in the developing OO, 5-dpf larvae were exposed to 10 μM CuSO_4_ (Figure 4) and processed for TUNEL labeling (Figures 4A–C). At 5 dpf, whole-mount control fish showed no cell death (Figure 4A). After exposure to 10 μM CuSO_4_, only the OOs were positive for TUNEL (Figure 4B, green, arrows). Quantification of TUNEL fluorescence in control and treated animals showed a statistically significant increase in fluorescence in the OOs of copper-treated animals (Figure 4C).

**Figure 5:**
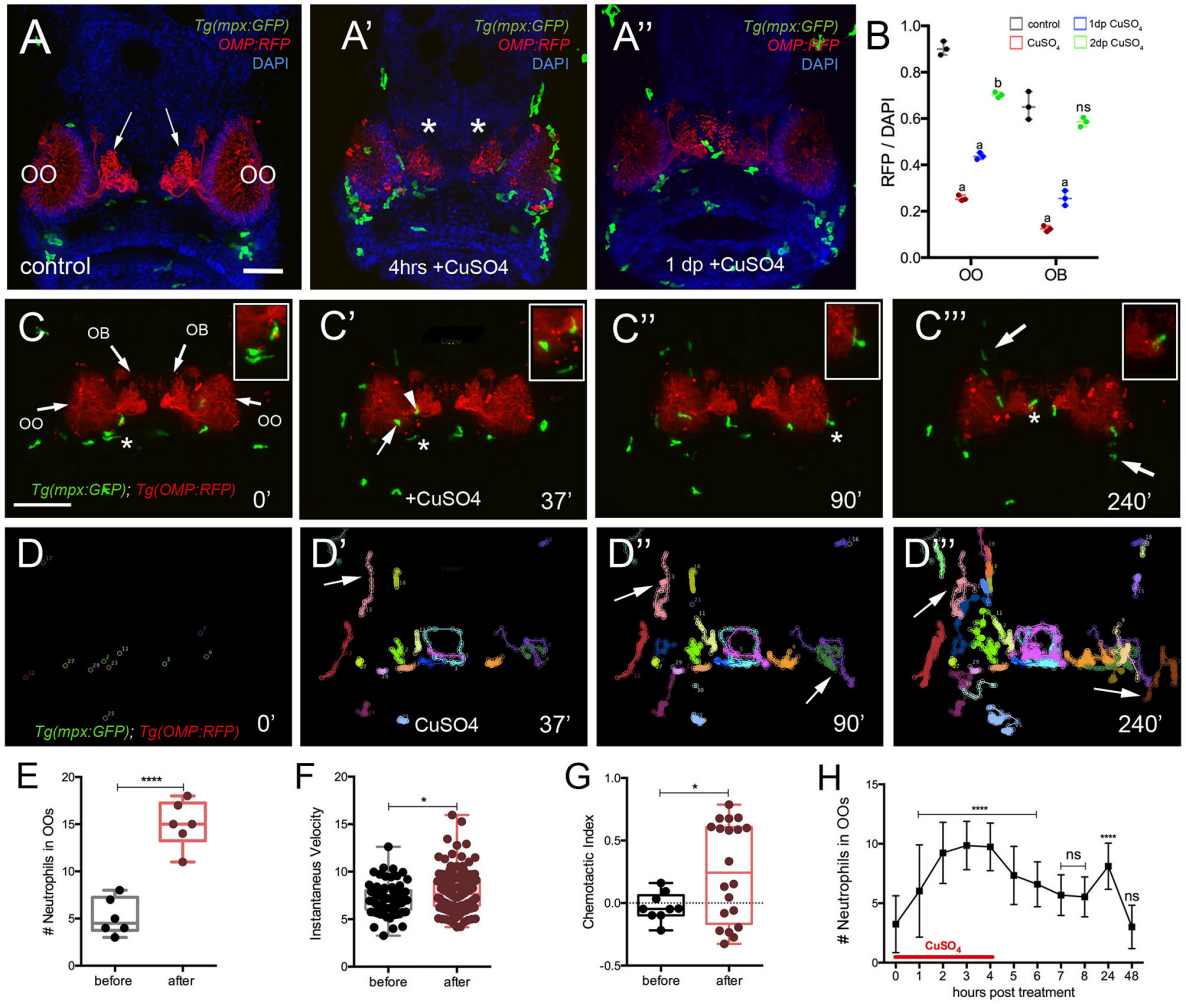
Exposure to copper induces migration of neutrophils. **(A-A″)** Frontal view of *Tg(mpx:GFP);Tg(OMP:RFP)* larva with OSNs (red) extending into OB. **(A)** Control. **(A′)** At 4 h post-copper exposure, there was an increase in neutrophils (green) in the OO and OSNs degenerated (red, asterisk). **(A″)** One day post treatment (dp) neutrophils decreased and the OSNs (red) were recovering. **(B)** Quantification of OSN (red fluorescence) in control (gray), copper-treated animals (red), 1 day (green bar) and 2 days posttreatment (blue bar). All fluorescence was normalized using DAPI (*n* = 3 larvae, two-way ANOVA, Tukey multiple-comparisons test, ****a = P < 0.0001, b = P < 0.01). All scale bars = 100 μm. **(C–C**^**‴**^**)** 5-dpf *Tg(mpx: GFP);Tg(OMP:RFP)* larva, frontal view. Imaging was initiated at time 0′. At 37′ **(C′)**, larvae were exposed to 10 μM of CuSO_4_, and imaging continued at times indicated (see Supplementary Video 1 for sequence taken every minute). Boxed areas: Neutrophils (green, asterisks) associated with olfactory organ (OO, **C–C″**) and OMP:RFP+ OSNs in olfactory bulb (red, asterisk, **C**^**‴**^). **(C″,C**^**‴**^**)** Arrows in **(C**^**‴**^**)** non-local neutrophils that do not enter the OO near the ON (see Supplementary Video 4). **(D–D**^**‴**^**)** Individual 2D-cell tracking of neutrophils before, during, and after copper exposure. Each color represents a different neutrophil. **(E)** Number of neutrophils within the OO before and after copper exposure: analysis of six videos from different animals. Unpaired *t*-test, P < 0.0001). **(F)** Speed of neutrophils before and after copper exposure (*n* = 30 neutrophils; Unpaired *t*-test, P < 0.05). **(G)** Chemotactic index of neutrophils before and after copper exposure (*n* = 20 neutrophils; Unpaired *t*-test, P < 0.05). **(H)** Time course of neutrophil movement to the OO (*n* = 48 larvae. ANOVA, Kruskal–Wallis test, P < 0.0001). Scale bar **C–C**^**‴**^ = 150 μm. Tracking was done using the ImageJ plugin, MTrackJ.

The olfactory sensory system has several sensory cell types, and the *Tg(OMP:RFP)* reporter line is expressed only in ciliated OSNs, the most abundant sensory neuron type in the OO. Because differential sensitivity has been reported for ciliated and microvillous OSNs, we visualized the microvillous OSNs using the *Tg(trpc2:GFP)* line combined with *Tg(lysc:DsRed)* to visualize neutrophils in red (Figures 4D,E). Quantification of pixel intensity changes for Trpc2:GFP+ fluorescence confirmed that, unlike ciliated OSNs (Figure 5), microvillous OSNs were largely unaffected by copper exposure (Figures 4D,E, green; Figure 4F, gray bar). For all experiments (Figures 4D,E), 25 larvae were processed and examined (control and copper-exposed). Of these, three different animals were analyzed from each treatment group. These results confirm that the damage caused by copper exposure is consistent and comparable with previous studies in zebrafish (Ma et al., 2018).

### *In vivo* Neutrophil Response to Cell Damage

To confirm whether copper-induced damage affected the ciliated OSNs and triggered a neutrophil response, we exposed 5-dpf *Tg(mpx:GFP); Tg(OMP:RFP*) to copper (Figures 5A–A″, OSNs: red, neutrophils: green) and assayed the changes in fluorescence in the OSNs (Figure 5B). After 4 h of copper exposure, neutrophils were found in the OO (Figure 5A′, green) and the OSNs degenerated as evidenced by loss of OMP:RFP fluorescence (Figure 5A′, red). Quantification of pixel intensity changes in OMP:RFP+ fluorescence confirmed that OSNs degenerated when scored immediately after copper exposure (Figure 5B, red bar). When scored at 24 h after exposure (Figure 5B, green bar) and 48 h after exposure, a steady increase in OMP:RFP fluorescence was observed where OSN fluorescence in the OB returned to pre–copper exposure levels (control, Figure 5A), indicating the OSNs had recovered (Figure 5C, blue bar). Analyses were performed both in the OO where the cell bodies of the OSNs are located and in the OB where the axons form their terminations (Figure 5A, arrows; Figure 5B, asterisks, respectively).

To better understand the dynamics of neutrophil response to OSN damage in the OO, we performed time lapse imaging in whole-mount preparations using a *Tg(OMP:RFP):Tg(mpx:GFP)* double transgenic line to visualize OSNs (Figures 5C–C^‴^; red) and neutrophils *in vivo* (Figures 5C–C^‴^; green; Supplementary Video 1). Before copper exposure, local neutrophils (defined as those associated with the OO prior to initiating the time lapse; Figure 5C, green) were associated with the OSNs at the margins of the OO (Figure 5C, red, asterisk indicates boxed area). During copper exposure, local neutrophils were associated with the OO (Figure 5C′, arrow) and ON (Figure 5C′, arrowhead, asterisk indicates boxed area). Subsequently, neutrophils in the OO (Figure 5C, asterisk indicates boxed area) were joined by neutrophils associated with the axons of OSNs in the OB (Figure 5C^‴^, asterisk indicates boxed area) and by patrolling non-local neutrophils (Figure 5C^‴^, arrows) in an apparent “swarming behavior” (Supplementary Video 1).

Analysis of the 2D path of individual neutrophils showed that copper exposure triggered the migration of non-local neutrophils from the dorsal and ventral sides of the head (Figures 5C^‴^,D′–D^‴^, arrows), and these entered the OO and OB regions via pathways separate from the ON (Figure 5C^‴^, arrows). During the time of copper exposure (4 h), the number of neutrophils increased from a pre-exposure average of 5.2 ± 1.7 to 15.0 ± 2.4 (Figure 5E, data from analysis of 6 different time lapse videos). The mean speed of neutrophils after copper exposure increased from a pre-exposure velocity of 7.1 ± 0.2 μm/min, to a post-exposure velocity of 7.8 ± 0.1 μm/min (Figure 5F, *n* = 30 neutrophils; one representative video). Both velocities were in the range of the reported 11 μm/min for randomly migrating neutrophils in the ventral region of the head of 3-dpf zebrafish larvae (Walters et al., 2010). Analysis of the CI (Figure 5G) showed a significant increase in orientation toward the OO [CI of −0.05 (range, −0.21 to 0.15) to 0.24 (from −0.33 to 0.78)] but with a separation of groups, reflecting different patterns of movement of local neutrophils, which moved within the OO vs. those of non-local populations, which appeared to circulate in and out of the OO region (Figures 5D′–D^‴^, arrows, G, red). The number of neutrophils in the OOs remained elevated in the continued presence of copper (9.8 ± 1.9) and decreased after washout (Figure 5H). A second increase is seen 24 h after copper treatment (8.1 ± 1.9; *n* = 48), which may be associated with the replacement of ciliated OSNs (Figures 5A–A″, B), which have a lifelong program of cell renewal that is distinct from damage-induced regeneration. The number of neutrophils then returned to baseline at the end of the second-day post-exposure (Figure 5H), a time when the OSNs have recovered (Figure 5B). Thus, consistent with previous reports, in juvenile zebrafish, damage triggers a rapid mobilization of neutrophils, and chemotaxis contributes to the migration of neutrophils to the site of damage (Mathias et al., 2006), which also correlates with the time course of neuronal regeneration.

### Macrophage Response to Cell Damage

**Figure 6:**
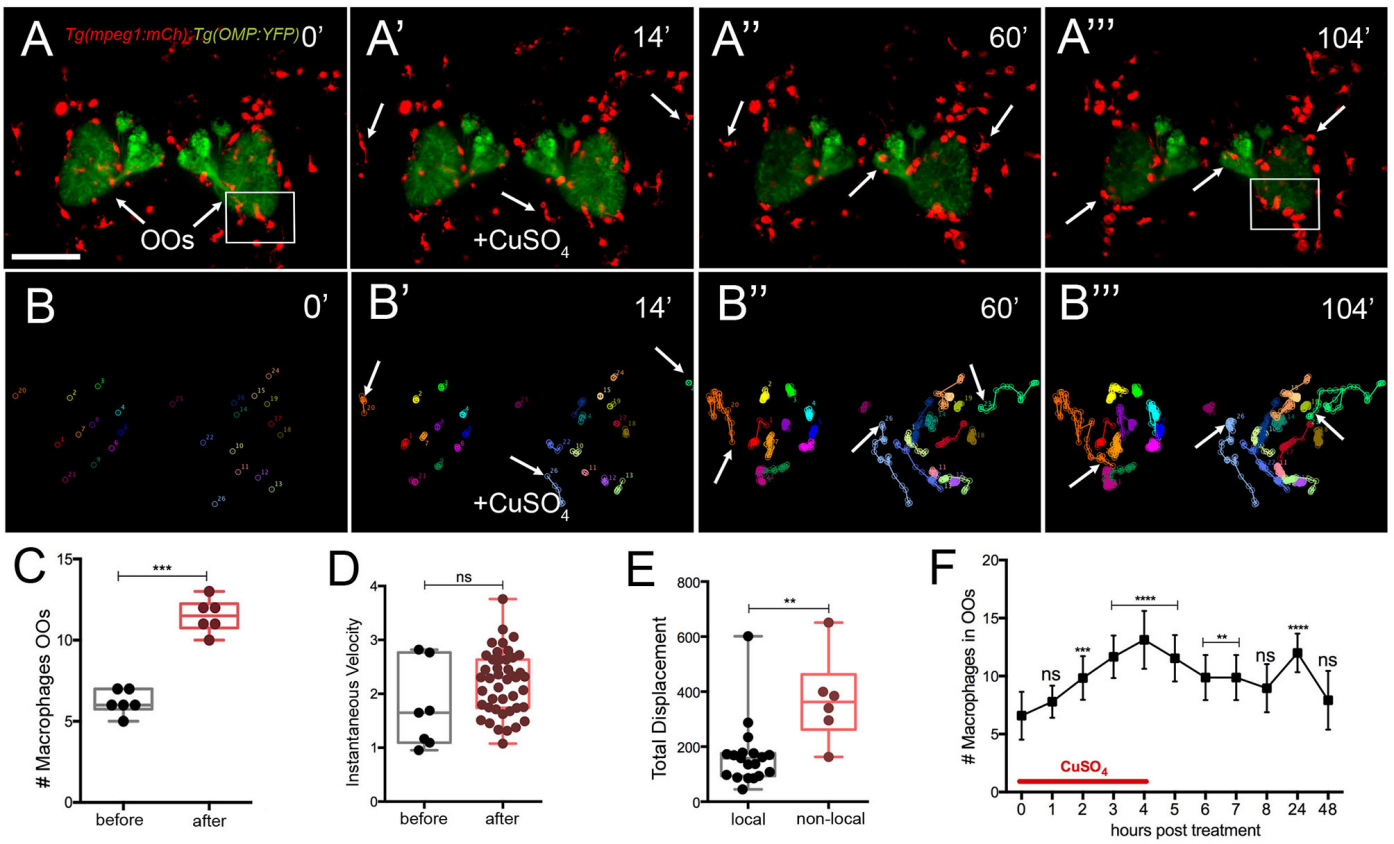
Exposure to copper induces migration response of macrophages in the olfactory organ. **(A-A**^**‴**^**)** 5-dpf *Tg(mpeg1:mCh);Tg(OMP:YFP)* larva frontal view. Imaging was initiated at time 0′. At 14′ **(A′)**, larvae were exposed to 10 μM of CuSO_4_ and imaging continued at times indicated (see Supplementary Video 2 for sequences taken every 2 min). **(A″, A**^**‴**^**)** Arrows in **(A**^**‴**^**)**: non-local macrophages that enter the OO (see Supplementary Video 2). **(B–B**^**‴**^**)** Individual 2D-cell tracking of macrophages associated with the OOs before, during, and after copper exposure. Each color represents a different macrophage. **(C)** Number of macrophages within the OO before and after copper exposure: analysis of three independent videos, (Unpaired *t*-test, P < 0.05). **(D)** Speed of macrophages before and after copper exposure (*n* = 50 macrophages, 1 time lapse; Unpaired *t*-test, P < 0.001). **(E)** Total displacement of local and non-local macrophages (Supplementary Video 2) during a 2-h time lapse (*n* = 51 macrophages, 16 local, 35 non-local; unpaired *t*-test, P < 0.0001). Scale bars: **A** = 150 μm. Tracking was done using the ImageJ plugin, MTrackJ. **(F)** Time course of macrophage movement to the OO (*n* = 24 larvae. ANOVA, Kruskal–Wallis test, P < 0.0001).

To better understand the dynamics of the macrophage response to OSN damage in the OO, we performed time lapse imaging in whole-mount preparations using a *Tg(mpeg1:mCh); Tg*(*OMP:RFP)* double transgenic line to visualize macrophages (Figures 6A–A″; red) and OSNs (Figures 6A–A^‴^; green; Supplementary Video 2). Before copper exposure, an extensive population of macrophages was found both associated with the OO (Figure 6A, green) and extending dorsal and ventral to the OOs (Figure 6A, red). In contrast to neutrophil migration induced during copper exposure, macrophages were closely associated with the OO (Figure 6A, arrows) and ON (Figure 6A). During copper exposure, macrophages in the OOs (Figure 6A, red) were joined by macrophages located outside the OOs (Figures 6A′,A″, arrows; Supplementary Video 2). Analysis of the 2D path of only the macrophages associated with the OOs showed that copper exposure resulted in a statistically significant increase in the number of macrophages in the OOs (Figures 6B,B^‴^,C), with specific non-local macrophages moving toward the OOs (Figures 6B′–B^‴^, arrows). These macrophages entered the OO and OB via pathways separate from the ON (Figure 6A^‴^, arrows). Similar to neutrophils, exposure to copper resulted in an increased number of macrophages (Figure 6C; 11.5 ± 0.4) associated with the OO when compared to the controls (Figure 6C; 6.2 ± 0.3; data from analysis of six independent time lapse videos). In contrast to neutrophil dynamics, no significant difference in instantaneous velocity of the macrophages was observed (Figure 6D, pre-exposure velocity of 1.7 ± 0.7 μm/min to a post-exposure velocity of 2.2 ± 0.6 μm/min; *n* = 26 macrophages; 1 video), and both velocities were significantly slower than that observed for neutrophils after exposure to copper (pre 7.1 ± 0.2 μm/min/post 7.8 ± 0.1 “μm/min). When quantifying the total displacement of the tracked macrophages, there was a significant difference between two groups of macrophages (Figure 6E): local macrophages that remained in close association with the OOs, moving <100 μm, and non-local (or wandering) macrophages that exceeded this total displacement (*n* = 51 macrophages, 16 local, 25 non-local, one representative time lapse). During copper exposure, there is a steady increase in macrophages associated with the OO (Figure 6F) that starts to decrease when copper is removed. Like neutrophils, 24 h post-exposure, there is a statistically significant increase of macrophages in the OOs that returns to baseline values at 48 h posttreatment (Figure 5F).

**Figure 7:**
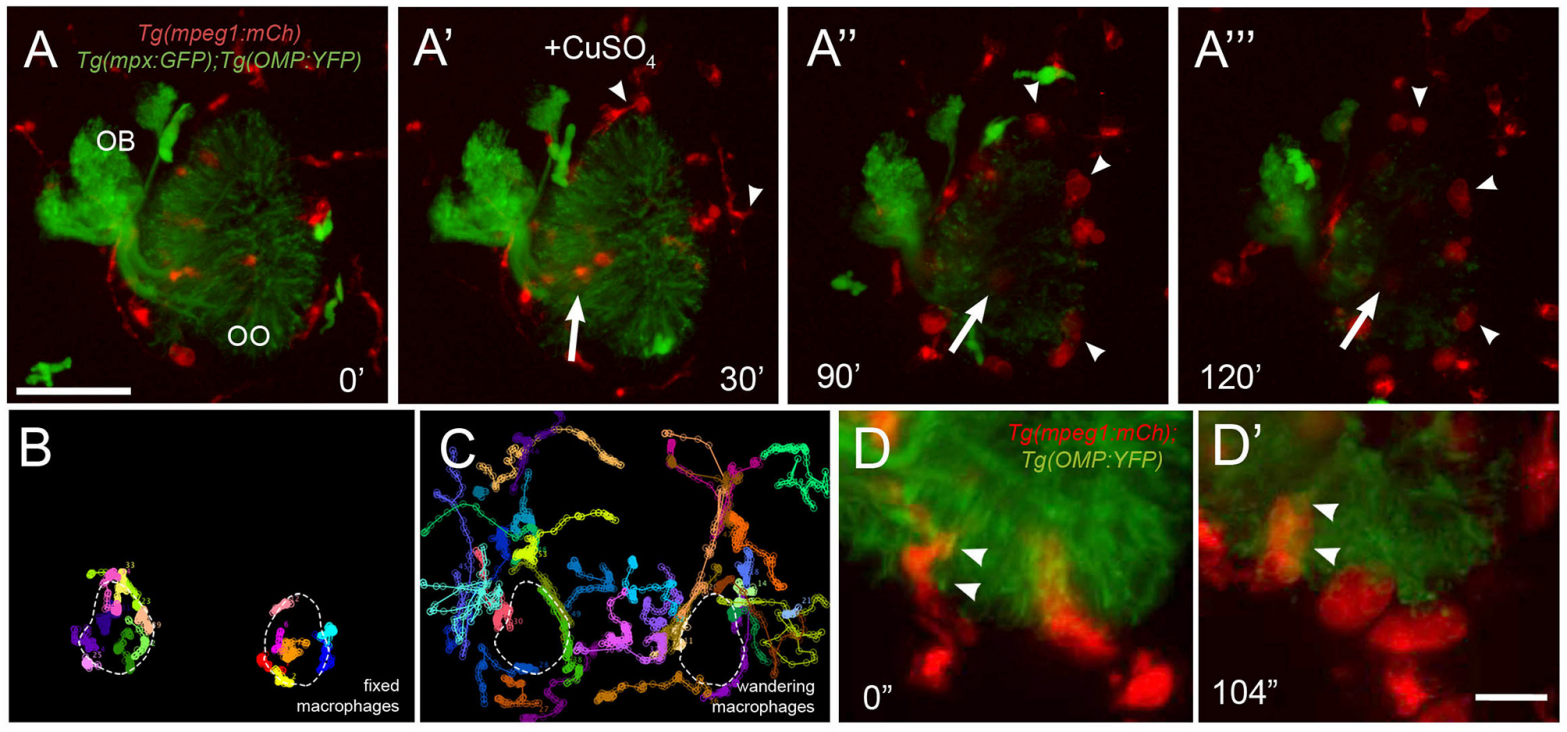
Exposure to copper reveals distinct classes of macrophages. **(A-A**^**‴**^**)** 5-dpf *Tg(mpeg1:mCh);Tg(mpx:GFP);Tg(OMP:YFP)* olfactory organ (OO) and imaging continued at times indicated (see Supplementary Video 3 for sequence taken every minute). **(A)** Imaging was initiated at time 0′. **(A′)** Larvae were exposed to 10 μM of CuSO_4_ at 30′. Individual cell tracking reveals the presence of **(B)** fixed and **(C)** wandering macrophages. **(D)** Before copper exposure (from boxed are in **A**), macrophages (red) are found associated with OSNs (green) in the ventral OO. **(D′)** After exposure to copper (from boxed are in Figure 6A^‴^), macrophages (mpeg1:mCh+, red) accumulate at the ventral–basal OO, in close association with degenerating OSNs (green, OMP:YFP+). Arrowheads indicate places where macrophages appeared to engulf degenerating OSNs (mpeg1:mCh+ and OMP:YFP+). Scale bars: **A-A**^**‴**^ = 75 μm, **D** = 25 μm.

To better understand the dynamics of macrophage movements in the OOs, we analyzed the movements of macrophages and neutrophils relative to copper-induced damage of the OSNs in *Tg(mpeg1:mCh):Tg(mpx:EGFP*):*Tg*(*OMP:YFP)* triple transgenic larvae (Figures 7A–C; Supplementary Video 3). As the OSNs degenerated, evidenced by the fading of green signal (Figures 7A–A^‴^), the macrophages associated with the OSNs (Figures 7A–A^‴^, arrow) and those at the perimeter of the OMP:YFP+ population (Figures 7A–A^‴^, arrowheads) swell over time (see Figures 6A′–A^‴^, red; Figures 7D,D′, red), potentially reflecting their role in phagocytosis of damaged OSNs. In further analysis of the macrophage movements using cell tracking, two distinct populations were observed: fixed macrophages (Figure 7B) (previously called local macrophages) that were always in the OOs, and “wandering” or non-local macrophages (Figure 7C) that were able to enter the OOs when damage occurs, but were patrolling the head before olfactory damage. Therefore, the local or fixed macrophages, observed using the *Tg(mpeg1:mCh)* line, were found in the OO and their behavior contrasts sharply with the wandering phenotype, perhaps reflecting different roles and subtypes of the phagocytic cells within the zebrafish head.

In response to copper-induced damage of the OSNs, both local and non-local macrophages changed their shape from a ramified-star–like shape (Figure 7D, red, arrowheads) to a rounded swollen morphology (Figure 7D′, red, arrowheads). In contrast to neutrophils, in response to copper, macrophages formed multiple vesicles and phagosome-like structures (Figures 7D,D′) (Peri and Nusslein-Volhard, 2008), which were observed engulfing the OMP:YFP+ degenerating OSNs (Figure 7D′, arrowheads). Thus, the macrophages associated with the OOs during early development are greater in number, respond more slowly to copper-induced damage, and show distinct phagocytic behaviors.

### Blood Lymphatic Vasculature and Neutrophil Migration

**Figure 8:**
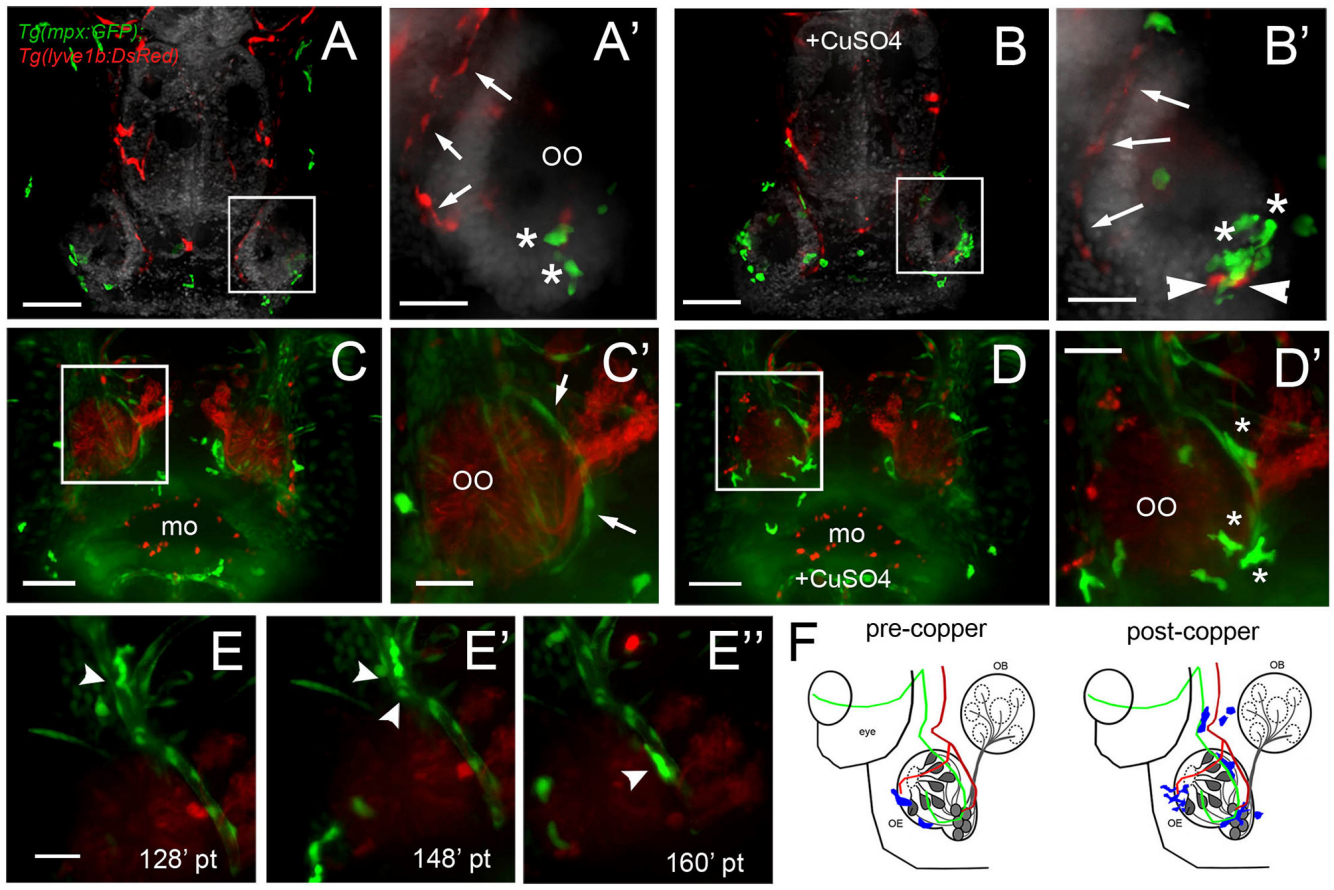
During copper exposure neutrophils migrate in association with blood vasculature to reach to the developing olfactory organ. **(A)** Frontal view of *Tg(mpx:gfp);Tg(lyve1b:*DsRed) 7 dpf larva. Boxed area **(A′)** with nasal LV (arrows, red) and neutrophils (green, asterisks). **(B)** Frontal view of *Tg(mpx:GFP);Tg(lyve1b:DsRed)* 7 dpf larva after 4 h of copper exposure, neutrophils (green, asterisks) associated with LV in ventral–lateral OO (arrowheads, red). Boxed area **(B′)** indicates cluster of neutrophils (asterisks, green). **(C–D′)** Images from Supplementary Video 4, *Tg(fli1a:EGFP)*;Tg(*gata1a:DeRed);Tg(mpx: GFP);Tg(OMP:RFP* (quadruple transgenic larva of 5 dpf, showing OSN and erythrocytes in red, and neutrophils and endothelial vasculature in green. **(C)** Before copper exposure. Boxed area is magnified in **(C′)**. Arrows indicate fli1a:EGFP+ branches that enclose the OO (Figure 2G′, NV, green). **(D)** After exposure to copper, more neutrophils are associated with the OO. **(D′)** Image from boxed area in **(D)**. Asterisks indicate neutrophils that have migrated to the OO on the BV (NV) and clustered around the medial edge of the OO. **(E–E″)** A polarized neutrophil crawling (arrowhead) along the NV to finally enter the OO near the ventral basal ON (**E″**, see Supplementary Video 5). Minutes are posttreatment (pt). **(F)** Summary: Schematic of nasal blood and lymphatic vasculature at 5 dpf before and after exposure to copper. OO: olfactory organ, OB: olfactory bulb. fli1a:EGFP (green) and lyve1b:DsRed (red), neutrophils (blue) migrate in response to copper exposure using the NV **(C′-D′)**, entering the OO near ventral ON exiting, and associating with ventral–lateral LV **(A′-B′)**. Scale bars: **(A–D)** = 100 μm; **(A′-D′,E–E″)** = 50 μm.

Because this was the first reported analysis of neutrophil responses in the OO, and our data on individual *in vivo* cell tracking suggested that neutrophils used preexisting pathways to reach the OOs in response to copper exposure, we further examined the neutrophil migration routes. To determine whether neutrophils migrated using BV and/or LV, we used *Tg(mpx:GFP); Tg(lyve1b:DsRed)* double-transgenic larvae to follow neutrophil movements associated with LV. Initially, there was no association of neutrophils with the developing rostral LV (data not shown), but in 7-dpf larvae, neutrophils were localized in the ventral–lateral OO (Figures 8A,A′, asterisks). After copper exposure the number of neutrophils increased (Figures 8A′,B′, green, asterisks) and were found associated with the lyve1b:DsRed+ branch of the ventral–lateral OO (Figures 8A′,B′, red, arrowheads; Figure 3G′, NL, red). To analyze the potential role of BV in neutrophil migration, we generated a quadruple reporter line *Tg(fli1a:EGFP);Tg(gata1a:DsRed);Tg(mpx:GFP);Tg(OMP:RFP)* allowing us to image in 5-dpf larvae *in vivo*: neutrophils (Figures 8C,C′,D,D″, green), the BV surrounding the OO (Figures 8C,C′,D,D″, green), the OSN (Figures 8C,C′,D,D″, red), and the erythrocytes within blood vessels (Figures 8C,C′,D,D″, green). Consistent with previous reports, we found that neutrophils showed a close association with the BV system in the developing embryo. In larvae exposed to copper, neutrophils moved along the NV on the medial side of the OO (Figures 8D,D′, asterisks, Supplementary Video 4). As the neutrophils migrate, they maintained intimate contact with the BV, often extending “feet” into the vasculature (Figures 8E–E″) as they moved (Supplementary Video 5). Thus, copper-induced damage to the developing OOs initiated neutrophil migration (Figure 8F, blue), which occurred along the medial NV (Figure 8F, green) at early stages and later included the ventral lateral OO associated with lyve1b:DsRed-positive LV branch (Figure 3G).

### Migration Route

**Figure 9:**
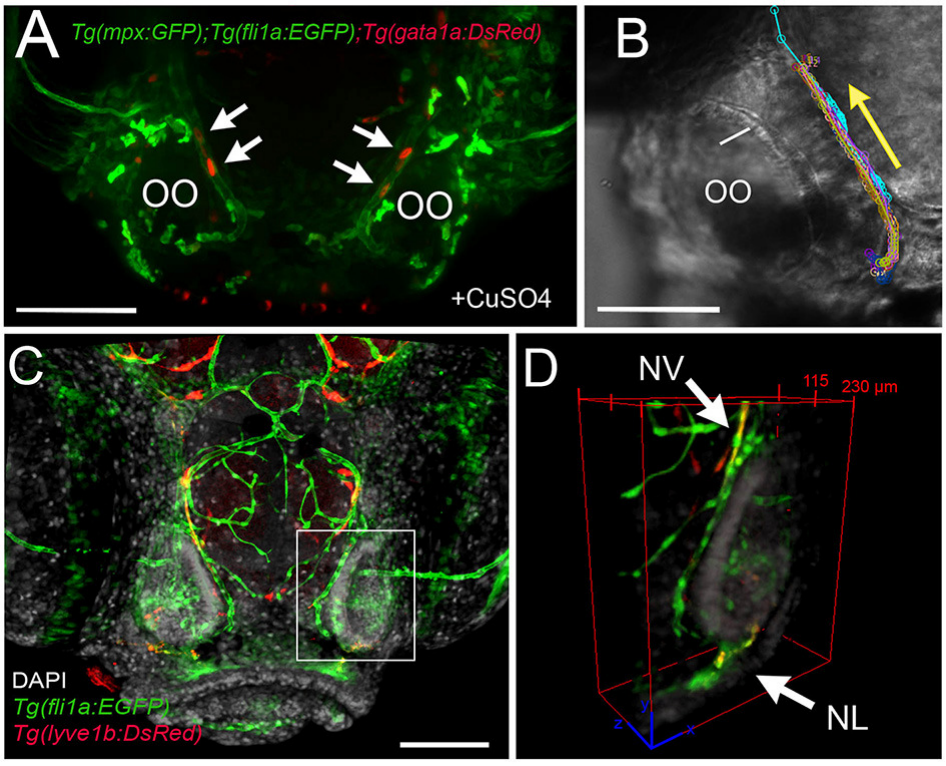
The nasal vein as the primary route to the olfactory organ during development. **(A)** *Tg(fli1a:EGFP);Tg(gata1a:DsRed)*;*Tg(mpx:GFP)* larva at 5 dpf. Erythrocytes (gata1:DsRed+, red, arrows) are observed within the nasal vein after copper exposure. **(B)** Tracking of blood flow of 10 erythrocytes circulating within the NV, whole-mount preparation in transmitted light (video of 2 min). Each color represents a different erythrocyte. Direction of movement is represented as a yellow arrow. **(C)** Laser confocal maximum projection of a 15-dpf *Tg(fli1a:EGFP); Tg(lyve1b:DsRed)* larva, DAPI (gray). **(D)** Three-dimensional orthogonal view generated from optic sections (boxed area in **C**), showing the NV (nasal vein, arrow) positive for fli1a:EGFP and lyve1b:DsRed. The NL (nasal lymphatics, arrow) is positive for lyve1b:DsRed and passes along the ventrolateral region of the OO. Total depth: 230 μm, 2-μm spacing. Scale bars: **(A,C)** = 100 μm, **(B)** = 50 μm.

Because the NA/NA are the primary routes for neutrophil migration to the OOs during early development and the classification as a vein or artery is unclear in the literature, we further analyzed the direction of blood flow in 5-dpf larvae. In *Tg(fli1a:EGFP);Tg(gata1a:DsRed);Tg(mpx:GFP)* triple transgenic larvae, we observed movement of erythrocytes (Figure 9A, red, arrows) in the NV and with a net direction from ventral to dorsal. Analysis of videos taken with transmitted light of whole-mount larvae *in vivo* confirmed the net direction as ventral to dorsal or “away” from the OO (Figure 9B, arrow). Furthermore, using *Tg(fli1a:EGFP);Tg(lyve1b:DsRed)* double-transgenic 15-dpf larvae, we confirmed that the nasal lymphatic branch (Figure 9D, NL, arrow, lyve1b:DsRed+) appeared in association with the nasal BV (Figure 9D, NV, arrow, fli1a:EGFP+ and lyve1b:DsRed+).

## Discussion

In this study, we examined the population of the developing OO by myeloid immune cells (neutrophils/macrophages) and their response to copper-induced damage. Key findings include the following: (1) both local and non-local neutrophils and macrophages are present in the developing OOs, where the local immune cells may play a role in the lifelong neurogenesis of the olfactory epithelia; (2) the appearance of the immune cells is correlated with the developing blood and LV of the OOs; (3) copper-induced damage triggers rapid but distinct responses from neutrophils and macrophages. Further studies are needed to determine the origin(s) of neutrophils and macrophages, as well as their different functions in developing and adult animals.

### Phagocytic Cell Populations in the Developing Olfactory Organ

Neutrophils are essential players in the innate immune system as they are the first cells that respond to tissue damage and infection by rapidly migrating to the site of injury (swarming) (de Oliveira et al., 2016). We first detected neutrophils in the OO at 3 dpf, well after the 30 hpf when functional macrophages and neutrophils are present (Herbomel et al., 1999; Le Guyader et al., 2008). Perhaps consistent with the necessity of a strong immune defense, the OO had significantly greater number of neutrophils than the mouth or ear (the eye had no neutrophils). In contrast to the neutrophils, macrophage populations in the developing olfactory OO and the eye were much larger than the other sensory systems, and there was no significant difference in numbers of macrophages found in the OOs and eyes. While little is known about macrophages in the larval retina, damage to the adult retina in zebrafish triggers the rapid accumulation of immune cells including local microglia and extraretinally derived macrophages (Mitchell et al., 2018). Microglia appear to play a role in the regulation of neurogenesis (Salter and Beggs, 2014), and macrophages may play a critical role in regeneration of sensory organs (Denans et al., 2019). At this time we cannot determine whether the macrophage population we have described in the OOs also includes precursors of microglia that, in zebrafish, arise from the primitive macrophages (Ferrero et al., 2018). The finding that both the eyes and the OOs have large macrophage populations, coupled with their anatomically unique peripheral extension of the meninges, which contain a diverse array of immune cells (Rua and McGavern, 2018), supports a model we proposed where the olfactory epithelia are more like the retina of the eye than placodal-derived structures (Whitlock, 2008; Torres-Paz and Whitlock, 2014; Torres-Paz et al., 2020). The presence of microglia in the peripheral olfactory sensory system would argue that the OO shares more characteristics with the CNS than peripheral nervous system, and we are currently investigating macrophage and microglia populations in the adult olfactory system.

### Neurogenic Response of Olfactory Organ

Unlike mammals, fish have the unique ability to maintain neurogenesis of sensory neurons throughout life. The exception to this difference is the olfactory epithelia where all vertebrates share the characteristic of ongoing sensory cell replacement (Bermingham-McDonogh and Reh, 2011). Copper, a heavy metal and pervasive environmental contaminant (Soller et al., 2005) is known to damage the olfactory sensory epithelia of fish, leading to loss of olfactory-driven behaviors (Sunderman, 2001; Baldwin et al., 2003; Matz and Krone, 2007), and to alter expression of genes involved in the olfactory signal transduction pathway in adult zebrafish (Tilton et al., 2008). Here we confirmed in larval zebrafish that exposure to copper resulted in OSN death (Lazzari et al., 2017; Ma et al., 2018) and the rapid recovery of the OSNs was accompanied by the influx of both neutrophils and macrophages.

Neutrophil movements we observed in the OOs are consistent with earlier studies in juvenile zebrafish, where damage triggers a rapid mobilization of neutrophils, and directed chemotaxis contributes to the migration of more neutrophils to the site of damage (Mathias et al., 2006). Moreover, it has also been shown that in response to wounding induced inflammation neutrophils move rapidly (15 μm/min) toward the wound, whereas macrophage migration velocity was significantly slower (Ellett et al., 2011; Dudek et al., 2020). Here we found similar results where neutrophils increased their velocity in response to damage, whereas macrophages showed a slower response. In contrast to wound healing responses induced by tail cutting, here we found that the OOs contain populations of local neutrophils and macrophages who were joined by non-local neutrophils and wandering macrophages in response to damage. This difference is most likely due to the unusual characteristics of the OSNs. In contrast to the tail wounding where the response is an inflammatory response in a tissue capable of regeneration, the OO is a tissue that has ongoing sensory neurogenesis over which is imposed neural damage induced by copper. Recent studies suggest that macrophages are involved in the repair of different neural tissue. In larval zebrafish, copper-induced hair cell damage in both the lateral line (Carrillo et al., 2016) and spinal cord transsection (Tsarouchas et al., 2018) resulted in the recruitment of neutrophils and macrophages to the injury site where macrophages were correlated with repair and regeneration of neural tissue. Because both macrophages and microglia are suggested to play a role in neurogenesis, as well as regeneration, the fixed or local macrophages we describe here (and potentially the local neutrophils) may play a role in the ongoing turnover of OSNs. Thus, the presence of both local and non-local neutrophils and macrophages in the developing OOs suggests a dual response where the local immune cells protect against external challenges, and non-local immune cells arrive only once damage is detected.

### Migration of Neutrophils and Macrophages

Studies in fish where a wounding response generated by tail and/or fin transsection (Ellett et al., 2011; Xu et al., 2012) have elucidated the role of macrophages and neutrophils in inflammation. In the wounding response, macrophages were found to patrol throughout the body, yet neutrophils were motile only in the head region of the larvae (Mathias et al., 2009; Deng et al., 2011; Ellett et al., 2011). While interstitial migration has been described for both neutrophils and macrophages (Barros-Becker et al., 2017), only neutrophils also use the blood–LV to migrate (Yoo et al., 2010; de Oliveira et al., 2016).

The LV has recently been “rediscovered” in the CNS of mammals (Aspelund et al., 2015; Louveau et al., 2015; Da Mesquita et al., 2018; Dolgin, 2020) and of zebrafish (Bower et al., 2017; van Lessen et al., 2017; Bower and Hogan, 2018), yet little is known about the development of the LV in the brain of vertebrates. Lymphatic endothelial cells are thought to arise from the BV system (Jung et al., 2017; Padberg et al., 2017); yet, to date, there are no detailed descriptions of the development of the BV and the LV in the olfactory sensory system. The development of the BV preceded the development of the LV in the OOs, and the primary route of neutrophil migration to the OOs was via the NVs whose development coincides with the first appearance of myeloid cells in the peripheral olfactory sensory system.

A fascinating question, brought to the fore by the current SARS-CoV2 pandemic, is how viruses gain access to the nervous system, and it is now apparent that the olfactory system is used by COVID-19 (coronavirus disease 2019) as an entry point to the nervous system (Desai and Oppenheimer, 2020; Divani et al., 2020). The study of the peripheral olfactory sensory system and the associated immune cells will allow us to better understand not only the rapid immune response to damage caused by toxic and infectious agents, but also how this neural immune interface may act as a host defense niche protecting the CNS.

## Conclusions

**Figure 10:**
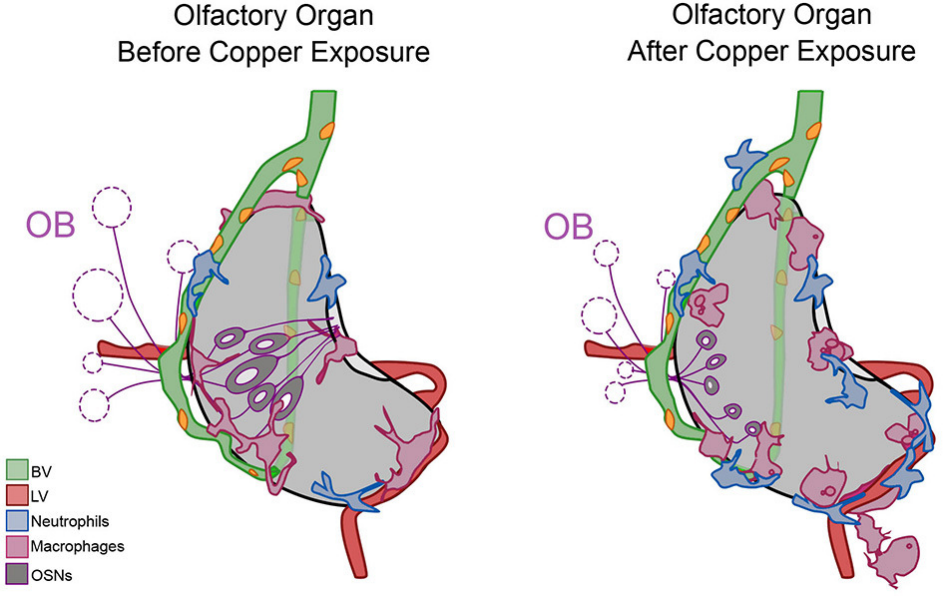
Summary of neutrophil and macrophage responses to copper-induced damage in the larval olfactory organ (OO). The blood vasculature (BV, green) wraps the olfactory organ (gray) and the lymphatic vasculature (LV, red) extends along the ventral posterior aspect. In untreated animals (before copper), there are local neutrophils (blue) and macrophages (pink) associated with the OO. In response to damage (after copper exposure) of the olfactory sensory neurons (OSNs, dark gray), non-local neutrophils and macrophages migrated to the OO. Neutrophils migrated in association with the BV, and both neutrophils and macrophages were seen associated with the LV. Macrophages changed to a more rounded morphology as they engulfed debris of dying OSNs.

During early development, at all times assayed, the OOs contain local populations of both neutrophils and macrophages, reminiscent of a potential host-defense niche described in other tissues where neutrophils are marginated (Yipp et al., 2017; Granton et al., 2018; Hidalgo et al., 2019) (Figure 10, Before Copper Exposure, blue). In response to damage non-local populations join local populations of neutrophils and macrophages as they mount a rapid immune response (Figure 10, After Copper Exposure, blue, pink). Neutrophils use the developing BV system (Figure 10, green) to access the OOs, and this may account for their greater velocity relative to macrophages.

## Data Availability Statement

The original contributions presented in the study are included in the article/Supplementary Material, further inquiries can be directed to the corresponding author/s.

## Ethics Statement

The animal study was reviewed and approved by El Comité Institucional de Bioética y Cuidado Animal, CIBICA (The Institutional Committee for Bioethics and Animal Care, CIBICA) Universidad de Valparaíso, Chile https://investigacion.uv.cl/cibica/quienes-somos-cibica/equipo-de-trabajo/ Dr. Pablo Muñoz Carvajal (Presidente) Bioquímico, Universidad de Santiago Doctor en Neurociencias, Universidad de Chile Profesor Adjunto de la Escuela de Medicina Facultad de Medicina cibica@uv.cl Edda Meléndez, Vicepresidenta Profesora de Música, Universidad de Playa Ancha Ingeniero en Gestión Industrial, Universidad Santa María Magíster en Educación, Universidad de Playa Ancha Profesora Auxiliar del Instituto de Filosofía Facultad de Humanidades Dr. Gonzalo Cruz. Miembro Titular Químico Farmacéutico Universidad de Valparaíso Doctor en Farmacología Universidad de Chile Profesor Auxiliar del Instituto de Fisiología Facultad de Ciencias Patricia Carreño González, Miembro Titular Químico Farmacéutico, Universidad de Chile Máster en Ingeniería de Alimentos, Universidad Politécnica de Valencia Profesora Titular de la Escuela de Química y Farmacia Facultad de Farmacia Enzo Seguel Avello, Miembro Titular Médico Veterinario, Universidad Iberoamericana de Ciencias y Tecnología Diplomado en Gestión de Calidad, Universidad Iberoamericana de Ciencias y Tecnología Veterinario Institucional Universidad de Valparaíso Miembro de Asociación Chilena en Ciencia y Tecnología de Animales de Laboratorio (ASOCHITAL) Claudia Delgado Acevedo, Miembro Suplente Médico Veterinario, Universidad de Chile Magíster en Ciencias Biomédicas, Universidad de Valparaíso Veterinaria Centro Interdisciplinario de Neurociencias, Universidad de Valparaiso Miembro de Asociación Chilena en Ciencia y Tecnología de Animales de Laboratorio (ASOCHITAL).

## Author Contributions

KEW: conceptualization of project, funding acquisition, investigation, formal analysis, project administration, supervision, validation, roles/writing original draft, and review & editing. MFP: investigation, implementation of experiments, formal analysis, methodology, resources, validation, visualization, and roles/writing–review & editing. All authors contributed to the article and approved the submitted version.

## Conflict of Interest

The authors declare that the research was conducted in the absence of any commercial or financial relationships that could be construed as a potential conflict of interest.

## Supplementary Material

Supplementary Video 1 Neutrophils mobilize in response to copper induced damage of the developing olfactory organ. Time lapse of *Tg(OMP:RFP0);Tg(mpx:GFP)* 5dpf larva, maximum projection of 150 μm depth (3 μm optical sections), taken every minute for 4 h. A final concentration of 10 μM of CuSO_4_ was added at minute 37 (Figure 5). Scale bar= 150 μm. Time in minutes is indicated in upper left corner. Arrowheads indicate local neutrophils (green). After treatment neutrophils where observed to swarm, cluster and even divide, next to OSNs (red).

Supplementary Video 2 Local and non-local macrophages respond to copper-induced damage in the olfactory organ. Time lapse of *mpeg1:mCh;mpx:EGFP* 5 dpf transgenic larva, maximum projection of 150 μm depth (3 μm optical sections), taken every 2 min for 1.7 h. A final concentration of 10 μM of CuSO_4_ was added at minute 14 (Figure 6). Scale bar= 150 μm. Time in minutes is indicated in upper left corner at the end of the time lapse macrophages are localized in the ventral and basal side of the olfactory organ, forming chain-like structures near branch of nasal lymphatics (Figures 3, 7).

Supplementary Video 3 Macrophages respond differently to copper exposure. Time lapse of *Tg(mpeg1:mCh);Tg(mpx:EGFP);Tg(OMP:YFP)* in 5 dpf larva, maximum projection of 150 μm depth (3 μm optical sections), taken every minute for 2 h. A final concentration of 10 μM of CuSO_4_ was added at minute 30 (Figure 7). Scale bar= 50 μm. Time in minutes is indicated in upper left corner. Local (or fixed) macrophages do not move, but displayed a swollen morphology after copper-induced damage.

Supplementary Video 4 Neutrophils move on the BV during early development. Time lapse of *Tg(fli1a:EGFP;Tg(gata1:DsRed);Tg(mpx:GFP);Tg(OMP:RFP)* quadruple transgenic larva at 5dpf, maximum projection of 300 μm depth with optical sections of 3 μm, taken every minute for 3.5 h. OSNs and erythrocytes in red; neutrophils and endothelial vasculature in green. A final concentration of 10 μM of CuSO_4_ was added at minute 37 (Figure 8). Scale= 150 μm. Time in minutes is indicated in upper left corner.

Supplementary Video 5 The nasal vein as route for neutrophil migration to the olfactory organ. Magnified image of the olfactory organ in a time lapse (35 min extract) of a *Tg(fli1a:EGFP;Tg(gata1:DsRed);Tg(mpx:GFP);Tg(OMP:RFP)* quadruple transgenic larva at 5 dpf, during copper exposure. Maximum projection of 300 μm depth, taken every minute. Scale bar= 50 μm. Time in minutes is indicated in upper left corner.

## References

[B1] Aspelund A. Antila S. Proulx S. T. Karlsen T. V. Karaman S. Detmar M.. (2015). A dural lymphatic vascular system that drains brain interstitial fluid and macromolecules. J. Exp. Med. 212, 991–999. 10.1084/jem.20142290 26077718 PMC4493418

[B2] Baldwin D. H. Sandahl J. F. Labenia J. S. Scholz N. L. (2003). Sublethal effects of copper on coho salmon: impacts on nonoverlapping receptor pathways in the peripheral olfactory nervous system. Environ. Toxicol. Chem. 22, 2266–2274. 10.1897/02-428 14551988

[B3] Barros-Becker F. Lam P. Y. Fisher R. Huttenlocher A. (2017). Live imaging reveals distinct modes of neutrophil and macrophage migration within interstitial tissues. J. Cell Sci. 130, 3801–3808. 10.1242/jcs.206128 28972134 PMC5702045

[B4] Bermingham-McDonogh O. Reh T. A. (2011). Regulated reprogramming in the regeneration of sensory receptor cells. Neuron 71, 389–405. 10.1016/j.neuron.2011.07.015 21835338 PMC4403668

[B5] Bower N. I. Hogan B. M. (2018). Brain drains: new insights into brain clearance pathways from lymphatic biology. J. Mol. Med. 96, 383–390. 10.1007/s00109-018-1634-9 29610928

[B6] Bower N. I. Koltowska K. Pichol-Thievend C. Virshup I. Paterson S. Lagendijk A. K.. (2017). Mural lymphatic endothelial cells regulate meningeal angiogenesis in the zebrafish. Nat. Neurosci. 20, 774–783. 10.1038/nn.4558 28459441

[B7] Calfun C. Dominguez C. Perez-Acle T. Whitlock K. E. (2016). Changes in olfactory receptor expression are correlated with odor exposure during early development in the zebrafish (*Danio rerio*). Chem. Senses 41, 301–312. 10.1093/chemse/bjw002 26892307

[B8] Carrillo S. A. Anguita-Salinas C. Peña O. A. Morales R. A. Muñoz-Sánchez S. Muñoz-Montecinos C.. (2016). Macrophage recruitment contributes to regeneration of mechanosensory hair cells in the zebrafish lateral line. J. Cell. Biochem. 117, 1880–1889. 10.1002/jcb.25487 26755079

[B9] Da Mesquita S. Fu Z. Kipnis J. (2018). The meningeal lymphatic system: a new player in neurophysiology. Neuron 100, 375–388. 10.1016/j.neuron.2018.09.022 30359603 PMC6268162

[B10] d'Alençon C. A. Peña O. A. Wittmann C. Gallardo V. E. Jones R. A. Loosli F.. (2010). A high-throughput chemically induced inflammation assay in zebrafish. BMC Biol. 8:151. 10.1186/1741-7007-8-151. 10.1186/1741-7007-8-151 21176202 PMC3022775

[B11] Davidson A. J. Zon L. I. (2004). The “definitive” (and “primitive”) guide to zebrafish hematopoiesis. Oncogene 23, 7233–7246. 10.1038/sj.onc.1207943 15378083

[B12] de Oliveira S. Rosowski E. E. Huttenlocher A. (2016). Neutrophil migration in infection and wound repair: going forward in reverse. Nat. Rev. Immunol. 16, 378–391. 10.1038/nri.2016.49 27231052 PMC5367630

[B13] Denans N. Baek S. Piotrowski T. (2019). Comparing sensory organs to define the path for hair cell regeneration. Annu. Rev. Cell Dev. Biol. 35, 567–589. 10.1146/annurev-cellbio-100818-125503 31553635

[B14] Deng Q. Yoo S. K. Cavnar P. J. Green J. M. Huttenlocher A. (2011). Dual roles for Rac2 in neutrophil motility and active retention in zebrafish hematopoietic tissue. Dev. Cell 21, 735–745. 10.1016/j.devcel.2011.07.013 22014524 PMC3199325

[B15] Desai M. Oppenheimer J. (2020). The importance of considering olfactory dysfunction during the COVID-19 pandemic and in clinical practice. J. Allergy Clin. Immunol. Pract. 28, 31188–31180. 10.1016/j.jaip.2020.10.036 33130145 PMC7598761

[B16] Divani A. A. Andalib S. Biller J. Napoli D. M. Moghimi N. Rubinos C. A. (2020). Central nervous system manifestations associated with COVID-19. Curr. Neurol. Neurosci. Rep. 20:60 10.1007/s11910-020-01079-7 33128130 PMC7599061

[B17] Dolgin E. (2020). Brain's drain. Nat. Biotechnol. 38, 258–262. 10.1038/s41587-020-0443-1 32066958

[B18] Dudek M. Rosowski A. Kozanecki M. Jaszczak M. Szymański W. Sharp M.. (2020). Microstructures manufactured in diamond by use of laser micromachining. Materials 13:1199. 10.3390/ma13051199 32155957 PMC7085071

[B19] Ellett F. Pase L. Hayman J. W. Andrianopoulos A. Lieschke G. J. (2011). mpeg1 promoter transgenes direct macrophage-lineage expression in zebrafish. Blood 117, e49–56. 10.1182/blood-2010-10-314120 21084707 PMC3056479

[B20] Ferrero G. Mahony C. B. Dupuis E. Yvernogeau L. Di Ruggiero E. Miserocchi M.. (2018). Embryonic microglia derive from primitive macrophages and are replaced by cmyb-dependent definitive microglia in zebrafish. Cell Rep. 24, 130–141. 10.1016/j.celrep.2018.05.066 29972775

[B21] Granton E. Kim J. H. Podstawka J. Yipp B. G. (2018). The lung microvasculature is a functional immune niche. Trends Immunol. 39, 890–899. 10.1016/j.it.2018.09.002 30253910

[B22] Hall C. Flores M. V. Storm T. Crosier K. Crosier P. (2007). The zebrafish lysozyme C promoter drives myeloid-specific expression in transgenic fish. BMC Dev. Biol. 7:42. 10.1186/1471-213X-7-42 17477879 PMC1877083

[B23] Harden M. V. Newton L. A. Lloyd R. C. Whitlock K. E. (2006). Olfactory imprinting is correlated with changes in gene expression in the olfactory epithelia of the zebrafish. J. Neurobiol. 66, 1452–1466. 10.1002/neu.20328 17013923

[B24] Herbomel P. Thisse B. Thisse C. (1999). Ontogeny and behaviour of early macrophages in the zebrafish embryo. Development 126, 3735–3745. 10433904 10.1242/dev.126.17.3735

[B25] Herbomel P. Thisse B. Thisse C. (2001). Zebrafish early macrophages colonize cephalic mesenchyme and developing brain, retina, and epidermis through a M-CSF receptor-dependent invasive process. Dev. Biol. 238, 274–288. 10.1006/dbio.2001.0393 11784010

[B26] Hernandez P. P. Undurraga C. Gallardo V. E. Mackenzie N. Allende M. L. Reyes A. E. (2011). Sublethal concentrations of waterborne copper induce cellular stress and cell death in zebrafish embryos and larvae. Biol. Res. 44, 7–15. 10.4067/S0716-97602011000100002 21720676

[B27] Hidalgo A. Chilvers E. R. Summers C. Koenderman L. (2019). The neutrophil life cycle. Trends Immunol. 40, 584–597. 10.1016/j.it.2019.04.013 31153737

[B28] Isogai S. Horiguchi M. Weinstein B. M. (2001). The vascular anatomy of the developing zebrafish: an atlas of embryonic and early larval development. Dev. Biol. 230, 278–301. 10.1006/dbio.2000.9995 11161578

[B29] Jung H. M. Castranova D. Swift M. R. Pham V. N. Venero Galanternik M. Isogai S.. (2017). Development of the larval lymphatic system in zebrafish. Development 144, 2070–2081. 10.1242/dev.145755 28506987 PMC5482986

[B30] Kimmel C. B. Ballard W. W. Kimmel S. R. Ullmann B. Schilling T. F. (1995). Stages of embryonic development of the zebrafish. Dev. Dyn. 203, 253–310. 10.1002/aja.1002030302 8589427

[B31] Lämmermann T. Afonso P. V. Angermann B. R. Wang J. M. Kastenmüller W. Parent C. A. (2013). Neutrophil swarms require LTB4 and integrins at sites of cell death *in vivo*. Nature 498, 371–375. 10.1038/nature12175 23708969 PMC3879961

[B32] Lawson N. D. Weinstein B. M. (2002). *In vivo* imaging of embryonic vascular development using transgenic zebrafish. Dev. Biol. 248, 307–318. 10.1006/dbio.2002.0711 12167406

[B33] Lazzari M. Bettini S. Milani L. Maurizii M. G. Franceschini V. (2017). Differential response of olfactory sensory neuron populations to copper ion exposure in zebrafish. Aquat. Toxicol. 183, 54–62. 10.1016/j.aquatox.2016.12.012 27992776

[B34] Le Guyader D. Redd M. J. Colucci-Guyon E. Murayama E. Kissa K. Briolat V.. (2008). Origins and unconventional behavior of neutrophils in developing zebrafish. Blood 111, 132–141. 10.1182/blood-2007-06-095398 17875807

[B35] Lieschke G. J. Oates A. C. Paw B. H. Thompson M. A. Hall N. E. Ward A. C.. (2002). Zebrafish SPI-1 (PU.1) marks a site of myeloid development independent of primitive erythropoiesis: implications for axial patterning. Dev. Biol. 246, 274–295. 10.1006/dbio.2002.0657 12051816

[B36] Louveau A. Smirnov I. Keyes T. J. Eccles J. D. Rouhani S. J. Peske J. D.. (2015). Structural and functional features of central nervous system lymphatic vessels. Nature 523, 337–341. 10.1038/nature14432 26030524 PMC4506234

[B37] Ma E. Y. Heffern K. Cheresh J. Gallagher E. P. (2018). Differential copper-induced death and regeneration of olfactory sensory neuron populations and neurobehavioral function in larval zebrafish. Neurotoxicology 69:141151. 10.1016/j.neuro.2018.10.002 30292653 PMC6944286

[B38] Masud S. Torraca V. Meijer A. H. (2017). Modeling infectious diseases in the context of a developing immune system. Curr. Top Dev. Biol. 124, 277–329. 10.1016/bs.ctdb.2016.10.006 28335862

[B39] Mathias J. R. Dodd M. E. Walters K. B. Yoo S. K. Ranheim E. A. Huttenlocher A. (2009). Characterization of zebrafish larval inflammatory macrophages. Dev. Comp. Immunol. 33, 1212–1217. 10.1016/j.dci.2009.07.003 19619578 PMC2742687

[B40] Mathias J. R. Perrin B. J. Liu T. X. Kanki J. Look A. T. Huttenlocher A. (2006). Resolution of inflammation by retrograde chemotaxis of neutrophils in transgenic zebrafish. J. Leukoc. Biol. 80, 1281–1288. 10.1189/jlb.0506346 16963624

[B41] Matz C. J. Krone P. H. (2007). Cell death, stress-responsive transgene activation, and deficits in the olfactory system of larval zebrafish following cadmium exposure. Environ. Sci. Technol. 41, 5143–5148. 10.1021/es070452c 17711236

[B42] McQuin C. Goodman A. Chernyshev V. Kamentsky L. Cimini B. A. Karhohs K. W.. (2018). CellProfiler 3.0: next-generation image processing for biology. PLoS Biol. 16:e2005970. 10.1371/journal.pbio.2005970 29969450 PMC6029841

[B43] Mescher A. L. Neff A. W. King M. W. (2017). Inflammation and immunity in organ regeneration. Dev. Comp. Immunol. 66, 98–110. 10.1016/j.dci.2016.02.015 26891614

[B44] Mitchell D. M. Lovel A. G. Stenkamp D. L. (2018). Dynamic changes in microglial and macrophage characteristics during degeneration and regeneration of the zebrafish retina. J. Neuroinflammation 15:163. 10.1186/s12974-018-1185-6 29804544 PMC5971432

[B45] Okuda K. S. Astin J. W. Misa J. P. Flores M. V. Crosier K. E. Crosier P. S. (2012). lyve1 expression reveals novel lymphatic vessels and new mechanisms for lymphatic vessel development in zebrafish. Development 139, 2381–2391. 10.1242/dev.077701 22627281 PMC4074227

[B46] Padberg Y. Schulte-Merker S. van Impel A. (2017). The lymphatic vasculature revisited-new developments in the zebrafish. Methods Cell Biol. 138, 221–238. 10.1016/bs.mcb.2016.11.001 28129845

[B47] Pägelow D. Chhatbar C. Beineke A. Liu X. Nerlich A. van Vorst K.. (2018). The olfactory epithelium as a port of entry in neonatal neurolisteriosis. Nat. Commun. 9:4269. 10.1038/s41467-018-06668-2 30323282 PMC6189187

[B48] Peri F. Nusslein-Volhard C. (2008). Live imaging of neuronal degradation by microglia reveals a role for v0-ATPase a1 in phagosomal fusion *in vivo*. Cell 133, 916–927. 10.1016/j.cell.2008.04.037 18510934

[B49] Renshaw S. A. Loynes C. A. Trushell D. M. Elworthy S. Ingham P. W. Whyte M. K. (2006). A transgenic zebrafish model of neutrophilic inflammation. Blood 108, 3976–3978. 10.1182/blood-2006-05-024075 16926288

[B50] Rua R. McGavern D. B. (2018). Advances in meningeal immunity. Trends Mol. Med. 24, 542–559. 10.1016/j.molmed.2018.04.003 29731353 PMC6044730

[B51] Sakano H. (2010). Neural map formation in the mouse olfactory system. Neuron 67, 530–542. 10.1016/j.neuron.2010.07.003 20797531

[B52] Salter M. W. Beggs S. (2014). Sublime microglia: expanding roles for the guardians of the CNS. Cell 158, 15–24. 10.1016/j.cell.2014.06.008 24995975

[B53] Sato Y. Miyasaka N. Yoshihara Y. (2005). Mutually exclusive glomerular innervation by two distinct types of olfactory sensory neurons revealed in transgenic zebrafish. J. Neurosci. 25, 4889–4897. 10.1523/JNEUROSCI.0679-05.2005 15901770 PMC6724860

[B54] Schindelin J. Arganda-Carreras I. Frise E. Kaynig V. Longair M. Pietzsch T.. (2012). Fiji: an open-source platform for biological-image analysis. Nat. Methods 9, 676–682. 10.1038/nmeth.2019 22743772 PMC3855844

[B55] Soller J. Stephenson J. Olivieri K. Downing J. Olivieri A. W. (2005). Evaluation of seasonal scale first flush pollutant loading and implications for urban runoff management. J. Environ. Manage 76, 309–318. 10.1016/j.jenvman.2004.12.007 15923077

[B56] Sunderman F. W. Jr. (2001). Nasal toxicity, carcinogenicity, and olfactory uptake of metals. Ann. Clin. Lab Sci. 31 3–24. 11314863

[B57] Tacchi L. Musharrafieh R. Larragoite E. T. Crossey K. Erhardt E. B. S.. (2014). Nasal immunity is an ancient arm of the mucosal immune system of vertebrates. Nat. Commun. 5:5205. 10.1038/ncomms6205 25335508 PMC4321879

[B58] Tierney K. B. Baldwin D. H. Hara T. J. Ross P. S. Scholz N. L. Kennedy C. J. (2010). Olfactory toxicity in fishes. Aquat. Toxicol. 96, 2–26. 10.1016/j.aquatox.2009.09.019 19931199

[B59] Tilton F. Tilton S. C. Bammler T. K. Beyer R. Farin F. Stapleton P. L.. (2008). Transcriptional biomarkers and mechanisms of copper-induced olfactory injury in zebrafish. Environ. Sci. Technol. 42, 9404–9411. 10.1021/es801636v 19174923 PMC3321378

[B60] Torres-Paz J. Tine E. M. Whitlock K. E. (2020). Dissecting the neural divide: a continuous neurectoderm gives rise to both the olfactory placode and olfactory bulb. Int. J. Dev. Biol. 10.1387/ijdb.200097kw. [Epub ahead of print]. 32930383

[B61] Torres-Paz J. Whitlock K. E. (2014). Olfactory sensory system develops from coordinated movements within the neural plate. Dev. Dyn. 243, 1619–1631. 10.1002/dvdy.24194 25255735 PMC4245031

[B62] Traver D. Paw B. H. Poss K. D. Penberthy W. T. Lin S. Zon L. I. (2003). Transplantation and *in vivo* imaging of multilineage engraftment in zebrafish bloodless mutants. Nat. Immunol. 4, 1238–1246. 10.1038/ni1007 14608381

[B63] Tsarouchas T. M. Wehner D. Cavone L. Munir T. Keatinge M. Lambertus M.. (2018). Dynamic control of proinflammatory cytokines Il-1β and Tnf-α by macrophages in zebrafish spinal cord regeneration. Nat. Commun. 9:4670. 10.1038/s41467-018-07036-w 30405119 PMC6220182

[B64] van Lessen M. Shibata-Germanos S. van Impel A. Hawkins T. A. Rihel J. Schulte-Merker S. (2017). Intracellular uptake of macromolecules by brain lymphatic endothelial cells during zebrafish embryonic development. Elife 6:e25932 10.7554/eLife.25932 28498105 PMC5457137

[B65] Walters K. B. Green J. M. Surfus J. C. Yoo S. K. Huttenlocher A. (2010). Live imaging of neutrophil motility in a zebrafish model of WHIM syndrome. Blood 116, 2803–2811. 10.1182/blood-2010-03-276972 20592249 PMC2974588

[B66] Westerfield M. (2007). The Zebrafish Book: A Guide for the Laboratory Use of Zebrafih (Danio rerio). Eugene, OR: University of Oregon Press.

[B67] Whitlock K. E. (2008). Developing a sense of scents: plasticity in olfactory placode formation. Brain Res. Bull. 75, 340–347. 10.1016/j.brainresbull.2007.10.054 18331896 PMC2443743

[B68] Whitlock K. E. (2015). The loss of scents: do defects in olfactory sensory neuron development underlie human disease? Birth Defects Res. C Embryo Today. 105, 114–125. 10.1002/bdrc.21094 26111003

[B69] Xu J. Du L. Wen Z. (2012). Myelopoiesis during zebrafish early development. J. Genet. Genomics 39, 435–442. 10.1016/j.jgg.2012.06.005 23021543

[B70] Xu J. Zhu L. He S. Wu Y. Jin W. Yu T.. (2015). Temporal-spatial resolution fate mapping reveals distinct origins for embryonic and adult microglia in zebrafish. Dev. Cell 34, 632–641. 10.1016/j.devcel.2015.08.018 26418294

[B71] Yipp B. G. Kim J. H. Lima R. Zbytnuik L. D. Petri B. Swanlund N. (2017). The lung is a host defense niche for immediate neutrophil-mediated vascular protection. Sci. Immunol. 2:eaam8929 10.1126/sciimmunol.aam8929 28626833 PMC5472445

[B72] Yoo S. K. Deng Q. Cavnar P. J. Wu Y. I. Hahn K. M. Huttenlocher A. (2010). Differential regulation of protrusion and polarity by PI3K during neutrophil motility in live zebrafish. Dev. Cell 18, 226–236. 10.1016/j.devcel.2009.11.015 20159593 PMC2824622

